# Towards autonomous analysis of chemical exchange saturation transfer experiments using deep neural networks

**DOI:** 10.1007/s10858-022-00395-z

**Published:** 2022-05-27

**Authors:** Gogulan Karunanithy, Tairan Yuwen, Lewis E. Kay, D. Flemming Hansen

**Affiliations:** 1 Division of Biosciences, Department of Structural and Molecular Biology, University College London, London, WC1E 6BT UK; 2 Department of Pharmaceutical Analysis and State Key Laboratory of Natural and Biomimetic Drugs, School of Pharmaceutical Sciences, Peking University, Beijing, 100191 China; 3 Department of Molecular Genetics, University of Toronto, Toronto, ON M5S 1A8 Canada; 4 Department of Chemistry, University of Toronto, Toronto, ON M5S 3H6 Canada; 5 Department of Biochemistry, University of Toronto, Toronto, ON M5S 1A8 Canada; 6 Program in Molecular Medicine, Hospital for Sick Children Research Institute, Toronto, ON M5G 0A4 Canada

**Keywords:** Artificial intelligence, NMR spectroscopy, Deep learning, Chemical exchange, CEST

## Abstract

Macromolecules often exchange between functional states on timescales that can be accessed with NMR spectroscopy and many NMR tools have been developed to characterise the kinetics and thermodynamics of the exchange processes, as well as the structure of the conformers that are involved. However, analysis of the NMR data that report on exchanging macromolecules often hinges on complex least-squares fitting procedures as well as human experience and intuition, which, in some cases, limits the widespread use of the methods. The applications of deep neural networks (DNNs) and artificial intelligence have increased significantly in the sciences, and recently, specifically, within the field of biomolecular NMR, where DNNs are now available for tasks such as the reconstruction of sparsely sampled spectra, peak picking, and virtual decoupling. Here we present a DNN for the analysis of chemical exchange saturation transfer (CEST) data reporting on two- or three-site chemical exchange involving sparse state lifetimes of between approximately 3–60 ms, the range most frequently observed via experiment. The work presented here focuses on the ^1^H CEST class of methods that are further complicated, in relation to applications to other nuclei, by anti-phase features. The developed DNNs accurately predict the chemical shifts of nuclei in the exchanging species directly from anti-phase ^1^H^N^ CEST profiles, along with an uncertainty associated with the predictions. The performance of the DNN was quantitatively assessed using both synthetic and experimental anti-phase CEST profiles. The assessments show that the DNN accurately determines chemical shifts and their associated uncertainties. The DNNs developed here do not contain any parameters for the end-user to adjust and the method therefore allows for autonomous analysis of complex NMR data that report on conformational exchange.

**Supplementary Information:**

The online version contains supplementary material available at 10.1007/s10858-022-00395-z.

## Introduction

Many functional aspects of a macromolecule can be understood from its time-averaged three-dimensional structure. However, often the functionality of these molecules depends on their ability to exchange between different conformational states. Thus, quantifying the interconversion between these states is an important first step towards understanding how these biomolecules work (Yang et al. 2003; Karplus and Kuriyan 2005; Boehr et al. 2006; Henzler-Wildman and Kern 2007; Faust et al. 2020; Xie et al. 2020; Wurm et al. 2021). When conformational exchange is present, there is often one major populated state, the ground state, and a set of transiently low-populated states that, despite their low populations and short lifetimes, often play crucial roles for function. Several NMR techniques are now available to characterise reaction dynamics and transiently populated states at atomic resolution, including, chemical exchange saturation transfer (CEST) (Ward et al. 2000; Zhou and Zijl 2006; Vallurupalli et al. 2012), dark-state exchange saturation transfer (DEST) (Bertini et al. 1999; Hansen and Led 2006; Fawzi et al. 2011), Carr-Purcell-Meiboom-Gill (CPMG) (Meiboom and Gill 1958; Loria et al. 1999; Tollinger et al. 2001) relaxation dispersion, and relaxation in the rotating frame (*R*_1ρ_, *R*_2ρ_) (Palmer and Massi 2006; Hansen et al. 2009; Chao and Byrd 2016). CEST-based methods, which report on conformational exchange involving sparse states with lifetimes ranging from approximately 3–60 ms, have expanded tremendously over the last decade and have provided invaluable insights into the function of macromolecules (Vallurupalli et al. 2017). However, although several tools are available for the analysis of NMR data reporting on conformational exchange, challenges do exist, particularly when the exchange deviates from a simple two-state model (Neudecker et al. 2006). For ^1^H CEST methods reporting on the exchange of amide-protons (Yuwen et al. 2017a) and methyl-protons (Yuwen et al. 2017b) analyses are further complicated by anti-phase features caused by the requirement to eliminate ^1^H-^1^H cross-relaxation effects, leading to broad lineshapes, with resolution significantly more limited than for ‘typical’ CEST profiles comprised of absorptive-like dips.

Deep learning and deep neural networks (DNNs) have led to huge advances in many fields of science, including computer vision and natural language processing, and the methodology is now a crucial component of many everyday technologies (LeCun et al. 2015). In supervised deep learning, DNNs are trained to map an input to a desired output, and once trained, these networks can perform analyses autonomously. Deep learning is particularly successful at extracting features in complex data (Goodfellow et al. 2016). It has been used for several years within the field of clinical magnetic resonance imaging (MRI) and some of the tools have already been approved by the FDA (Chaudhari et al. 2021) for image enhancement and classification. Within biomolecular NMR there has been a surge in applications of DNNs over the last couple of years, and networks are now available for the reconstruction of sparsely sampled spectra (Hansen 2019; Luo et al. 2020; Qu et al. 2020; Karunanithy and Hansen 2021), peak picking (Klukowski et al. 2018), estimating initial fitting parameters (Beckwith et al. 2021), and virtual decoupling (Karunanithy et al. 2021).

A key hurdle with many machine learning applications is that training robust models requires a large amount of curated training data. The in-depth understanding of the theory behind biomolecular NMR and the ability to simulate even complex NMR experiments means that the required amount of realistic training data can be generated synthetically. Importantly, it has now become clear that DNNs trained on fully synthetic data show robust performance on experimental data (Hansen 2019; Karunanithy and Hansen 2021; Karunanithy et al. 2021), which allows for sophisticated DNNs to be developed for the transformation and analysis of NMR spectra.

Overall, there is enormous potential for the development of deep learning approaches for the general analysis of NMR data and in particular for experiments reporting on conformational exchange. Below we have designed and trained DNNs to extract chemical shifts from the notably complex amide-proton anti-phase CEST experiment. The DNNs were trained solely on synthetically generated CEST profiles and are able to extract accurate chemical shifts of exchanging species as well as their uncertainties, thereby demonstrating that NMR data reporting on conformational exchange can be analysed autonomously using deep neural networks.

## Methods

### Deep neural network architectures

Figure S2 shows the architecture for the DNN used to transform time-domain anti-phase CEST profiles into time-domain in-phase CEST profiles, DNN_TR_. This architecture is built from two modules, a module akin to a block in the FID-Net architecture (Karunanithy and Hansen 2021) and a modified LSTM module (Hansen 2019). The reason for this choice was that the main objective for the DNN is to ‘decouple’ anti-phase CEST profiles, which we have recently shown can be accomplished by the FID-Net architecture (Karunanithy and Hansen 2021). The python code for generating the model architecture in Tensorflow/Keras is provided in Supporting Material and can be downloaded from GitHub. The input to the DNN consists of two vectors of size 2 × 65 = 130. The first vector, **cest**_AP_(*t*) = **c**_0_ holds the zero-filled real Fourier transform (real and imaginary components) of the antiphase CEST profile and the second vector holds the time-points associated with the first vector, **t**_0_. The output of the network is the in-phase CEST profile, sampled at 128 offsets. The network contained 3,782,423 trainable parameters.

The second DNN, DNN_CS_, used to determine chemical shifts and their confidences was built using a densely connected convolutional neural network architecture (Huang et al. 2016), Fig. S4. The input for the network is the output from the first transformation described above, that is, frequency domain data describing the in-phase CEST profile, **cest**_IP_(ω), a vector of 128 real points. In its current form, the network detects a maximum of three chemical shifts as well as their confidences and the output of the network is therefore a 3 × 2 tensor, whose elements comprise three chemical shifts and their confidence values. Overall, the network has 1,591,526 trainable parameters. The python code for generating the model in Tensorflow/Keras is provided in Supporting Material and can also be downloaded from GitHub.

### Training the deep neural networks

The first DNN, DNN_TR_, was trained on 15 × 10^6^ anti-phase CEST profiles over 1500 *epochs*, where the range of training data is detailed in Table 1. An *epoch* refers to a single cycle of training of the neural network with training data. The training data was generated on-the-fly using code written in python and using functions from the Tensorflow and numpy libraries. To obtain smooth simulated CEST profiles, similar to those generated by experiment, previous simulations have used a distribution of *B*_1_ fields or other dephasing methods (Vallurupalli et al. 2012). Here the dephasing was achieved by only retaining the eigenvectors of the Liouvillian corresponding to real eigenvalues in the propagator. Thus, if **L** is the matrix describing the Liouvillian, under which the spin-system evolves during the CEST period, then the eigenvalues and eigenvectors of **L** are initially found: **L Λ** = **Λ D**, where **Λ** is a matrix of eigenvectors and **D** is a diagonal matrix of eigenvalues. The submatrix of **D** that holds the real eigenvalues is denoted **D**_re_ and the matrix holding the eigenvectors corresponding to the real eigenvalues is denoted **Λ**_re_. Propagation of the spin-system is carried out with the propagator, **Λ**_re_ exp(− *T*_ex_**D**_re_) **Λ**^−1^_re_. As an example, for a simple Liouvillian, **L**, represented by a 3 × 3 matrix in the basis set of the three product operators, *I*_x_, *I*_y_, and *I*_z_ there is typically only one real eigenvalue. After an eigendecomposition of **L**, the matrix holding the eigenvectors, **Λ**, and the diagonal matrix holding the eigenvalues, **D**, are 3 × 3 matrices. The submatrix **Λ**_re_ has dimensions 3 × 1, **D**_re_, is a 1 × 1 matrix, and **Λ**^−1^_re_ is a 1 × 3 matrix. Thus, **Λ**_re_ **D**_re_ **Λ**_re_^−1^ produces a 3 × 3 matrix that is the projection of the original Liouvillian onto the space spanned by the real eigensystem and **Λ**_re_ exp(− *T*_ex_**D**_re_) **Λ**_re_^−1^ is the propagator corresponding only to the real eigensystem. For the code written with the Tensorflow library functions, where sizes of matrices should remain constant, the dephasing is achieved by multiplying any eigenvalue that has an imaginary part larger than 10^–3^ by 10^9^, which means that evolutions caused by non-real eigenvalues are eliminated within nanoseconds.

**Table 1:** Parameters used to generate training data

*Experimental parameters*	
B_0_	{14.1 T, 16.4 T, 18.8 T, 21.1 T, 23.5 T}
B_1_	15–50 Hz
Range of offset points	3.4 ppm
Sampled points	50–128
Inter-scan delay	0.5 s
CEST delay, T_ex_	0.4 s
*Parameters reporting on the spin system*	
Rotation correlation, τ_M_, used to calculate all relaxation rates of the ground state	3–20 ns
^1^H-^15^N scalar coupling, ^1^J_HN_	− 91 to − 95 Hz
Micro-second exchange contribution added to all states, R_ex_	Absolute value of a normal distribution: μ = 1.0 s^−1^, σ = 2.0 s^−1^
R_2,H_(E_1_) − R_2,H_(G) and R_2,H_(E_2_) − R_2,H_(G)	Normal distribution: μ = 0.0 s^−1^, σ = 2.0 s^−1^
Chemical shifts ω_G_, ω_E1_, and ω_E2_	Uniform distribution over the full sweep width
*Chemical exchange*	
Probability of three-site exchange	25%
k_ex,GE_, k_ex,GE1_, k_ex,GE2_	10–300 s^−1^
p_E_, p_E1_, p_E2_	0.01–0.15

The anti-phase CEST profiles were then obtained by propagating the Liouvillian over the first INEPT and the CEST element in the anti-phase ^1^H^N^ pulse sequence. For each anti-phase CEST profile an in-phase CEST profile was also generated by setting ^1^*J*_HN_ = 0 Hz and integrating the Liouvillian over the CEST element (Vallurupalli et al. 2012). The stochastic ADAM (Kingma and Ba 2014) optimiser was employed with standard parameters and an adaptive learning rate calculated as $0.0004 \times \left( {L_{{{\text{freq}}}} + L_{{{\text{uncer}}}} } \right)^{3/4}$ (final learning rate of 10^–6^). A batch size of 256 was used throughout the training and random gaussian noise was added with a standard deviation of 0.01 of the maximum value of each anti-phase CEST profile.

After training the DNN_TR_ network the DNN_CS_ network was trained. The input data for training the DNN_CS_ network was obtained from output of the trained DNN_TR_ network. Random gaussian noise with a standard deviation between 0.001 and 0.04 of the maximum value of each anti-phase CEST profile was added to anti-phase CEST profiles before these were transformed with the DNN_TR_ network. A total of 1.5 × 10^7^ CEST profiles were used for training, which was done over 110 *epochs*, with a batch size of 128. The stochastic ADAM (Kingma and Ba 2014) optimiser with standard parameters and a learning rate of 3.3 × 10^–4^ was used.

Initial training was carried out using a desktop computer (Intel Core I7-6900 K, 3.2 GHz, 64 GB RAM), equipped with an NVIDIA GeForce GTX 1080 TI GPU graphics card and subsequent training carried out using the CAMP cluster (NVIDIA Tesla V100 GPU). Although the training of the two DNNs has benefitted from access to nodes with GPUs, using the trained DNNs to transform new (experimental) data does not require high-end computational nodes or GPUs. As an example, the full set of *ca.* 140 ^1^H anti-phase CEST profiles from L99A T4 Lysozyme, Fig. 5, can be transformed with both DNN_TR_ and DNN_CS_ in less than 2 min on a standard laptop using only the CPU (Intel i7-6700 CPU).

### Experimental amide-proton CEST data

A 1.5 mM U-[^15^N, ^2^H] L99A T4L sample produced as described previously (Bouvignies et al. 2011) and dissolved in 50 mM sodium phosphate, 25 mM NaCl, 2 mM EDTA, 2 mM NaN_3_, pH 5.5, 90%H_2_O/10%D_2_O was used to record the anti-phase ^1^H^N^ CEST experiments. L99A T4L anti-phase ^1^H^N^ CEST experiments were performed as described previously (Yuwen et al. 2017a). Briefly, the experiments were measured on a 800 MHz Bruker spectrometer equipped with an x, y, z-gradient cryogenically cooled probe. ^1^H^N^-CEST measurements were performed with a *B*_1_ field of 30.5 Hz at 282 K using a CEST delay of *T*_ex_ = 400 ms. A range of ^1^H offsets on a regular grid from 6.5 to 9.5 ppm was used, with step sizes of 30 Hz. An additional reference 2D dataset was obtained by setting the *B*_*1*_ offset to − 12 kHz.

A 1.35 mM sample of [U-^15^N,^2^H; Ileδ_1_-^13^CHD_2_; Leu, Val-^13^CHD_2_/^13^CHD_2_; Met-^13^CHD_2_] G48A Fyn SH3 domain was prepared as described previously (Yuwen et al. 2017a). The sample was dissolved in 50 mM sodium phosphate, 0.2 mM EDTA, 0.05% NaN_3_, pH 7.0, 90% H_2_O/10% D_2_O. ^1^H^N^ CEST experiments were measured for the G48A Fyn SH3 domain using a 600 MHz Bruker spectrometer at 285 K (x, y, z-gradient cryogenically cooled probe). The ^1^H^N^ CEST datasets were recorded as described previously (Yuwen et al. 2017a); specifically, a pair of datasets was recorded using *B*_1_ fields of 26.7 Hz and 42.0 Hz. A CEST delay of *T*_ex_ = 400 ms was used and *B*_1_ offsets between 5.5 and 10.5 ppm with step sizes of 25 Hz (*B*_1_ = 26.7 Hz) or 40 Hz (*B*_1_ = 42 Hz) were recorded. In addition, a 2D reference dataset was obtained with a *B*_1_ offset of − 12 kHz that is equivalent to setting *B*_1_ = 0 Hz.

## Results and discussion

**Fig. 1:**
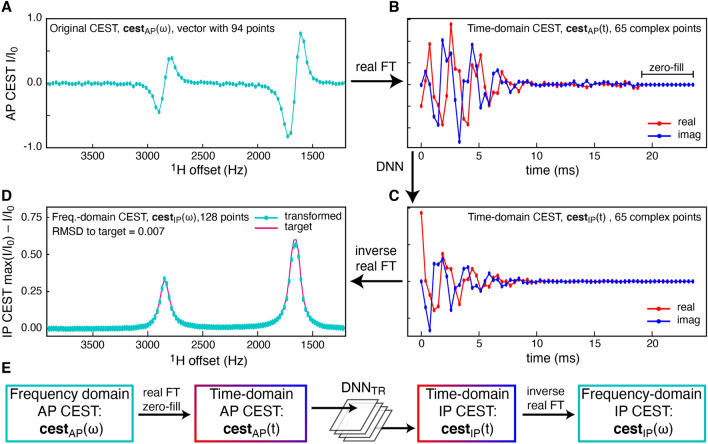
Transformation of an amide-proton CEST profile from anti-phase to in-phase. Initially the input anti-phase CEST profile (**A**) is transformed with a real Fourier transform to give the time-domain CEST profile, followed by zero-filling (an additional 17 complex points) to generate a time-domain profile of 65 complex points (**B**), independent of the size of the original CEST profile. The DNN_TR_ network decouples the time-domain anti-phase CEST profile to give (**C**), which is transformed with an inverse real Fourier transform to give the final in-phase CEST profile in (**D**). **E** Schematic representation of the transformation from anti-phase CEST profiles to in-phase CEST profiles with a fixed size

Chemical exchange saturation transfer profiles are normally visualised and analysed as, *I*(*ω*_offset_)/*I*_0_, where *I*(*ω*_offset_) is the intensity observed for a given site when a weak radio-frequency pulse (*B*_1_) is applied at a frequency of *ω*_offset_, and *I*_0_ is the corresponding intensity with no *B*_1_ pulse applied. A feature of standard CEST profiles is that they resemble inverted one-dimensional NMR spectra, where the ‘dips’ are centered at the chemical shifts of the exchanging species. Thus, the related CEST profile, max(*I*/*I*_0_) − *I*/*I*_0_, resembles a simple NMR spectrum and its real Fourier transform therefore resembles an FID. Analysis of the CEST profiles with DNNs shown below first involved transformation of the data into the time domain, through a real Fourier transform, Fig. 1A and B. It should be noted that for a real Fourier transform, or equivalently a discrete Fourier transform of pure real data (*N* data points), the output is Hermitian-symmetric and approximately half [*N*/2 − 1 for even *N* and (*N* − 1)/2 for odd *N*] of the points are therefore redundant, see Supporting Material and Fig. S1.

To show the strength of the developed DNNs for the analysis of CEST data, we consider the amide-proton anti-phase CEST (Yuwen et al. 2017a), whose profiles are complicated relative to those generated by other CEST experiments since the ‘dips’ are anti-phase in nature (i.e., multiplet components from the scalar coupling between one-bond ^1^H-X spins are of opposite phase). These CEST profiles are challenging to analyse primarily because the chemical shifts may not be easily accessible directly from the profiles. To facilitate the analysis of amide-proton CEST profiles the overall process is divided into two tasks, each with their own optimal DNN. The first DNN, DNN_TR_, transforms each anti-phase CEST profile into a ‘classical’ profile, where the doublet nature of the dips are eliminated, thereby improving resolution, and also upsamples the profile to a fixed number of points in the CEST dimension. The second DNN, DNN_CS_, then determines the ^1^H chemical shifts for each of the exchanging species and an associated confidence in the shift values.

### A deep neural network for the transformation of amide-proton CEST profiles

It was recently shown how each of the hidden layers of a simple DNN can be mapped to specific mathematical transformations (Amey et al. 2021). Such an approach is naturally highly attractive in order to design DNNs for new challenges and to understand their strengths and weaknesses. However, with the large size of recent networks developed to analyse and transform NMR data, our focus here is on employing architectures that have been shown recently to work well for related tasks. We have previously developed DNNs using the FID-Net architecture (Karunanithy and Hansen 2021) to decouple and analyse NMR spectra (Karunanithy and Hansen 2021; Karunanithy et al. 2021) by using FIDs as input. Since amide-proton anti-phase CEST profiles resemble anti-phase one-dimensional NMR spectra, our rationale was that a DNN similar to FID-Net can be trained to transform anti-phase CEST profiles into ‘decoupled’ standard CEST profiles. Thus, the DNN_TR_ architecture used was built of two modules, a module akin to a block in the FID-Net architecture (Karunanithy and Hansen 2021) and a modified long short-term memory (LSTM) module (Hansen 2019). The architecture is described in detail in Supporting Material, Fig. S2, where the python code for generating the model in Tensorflow/Keras (Chollet 2015; Abadi et al. 2016) is also provided. The theory for spin-evolution during CEST experiments is well-established (Helgstrand et al. 2000; Hansen et al. 2008; Vallurupalli et al. 2012), and synthetic training data can therefore easily be generated by propagating the Liouvillian over the desired element.

The first DNN, referred to as DNN_TR_, was trained to transform an input amide-proton anti-phase CEST profile to the hypothetical CEST profile of an isolated ^1^H spin, with ^1^*J*_HN_ = 0 Hz, Fig. 1. Thus, DNN_TR_ decouples the anti-phase amide proton CEST profile and upsamples it to 128 points. The upsampling to a constant size, in this case 128 real points, makes the prediction of chemical shifts with a second DNN feasible, since DNNs are typically trained with a constant size of the input and output data (see below). A maximum of three exchanging states was assumed and only the forked three-site exchange model was used to generate the data, that is, E_1_ ⇌ G ⇌ E_2_, where E_1_ and E_2_ are sparsely populated states. For 75% of the training data the population of E_2_ was set to zero. Because of the strong correlation between CEST data reporting on different three-site exchange models, for example, E_1_ ⇌ G ⇌ E_2_ *versus* G ⇌ E_1_ ⇌ E_2_, it is anticipated that DNN_TR_ will robustly transform anti-phase CEST profiles derived from any three-site exchange process. Briefly, DNN_TR_ was trained on 15 × 10^6^ CEST profiles, where the range of training data is indicated in Table 1. The loss function was calculated from the mean-squared-error between the transformed in-phase CEST profile and the target function, see Fig. 1D. The network was trained to a normalised mean-squared-error (MSE) of 4 × 10^–4^ and a mean-absolute-error (MAE) of 0.01.

**Fig. 2:**
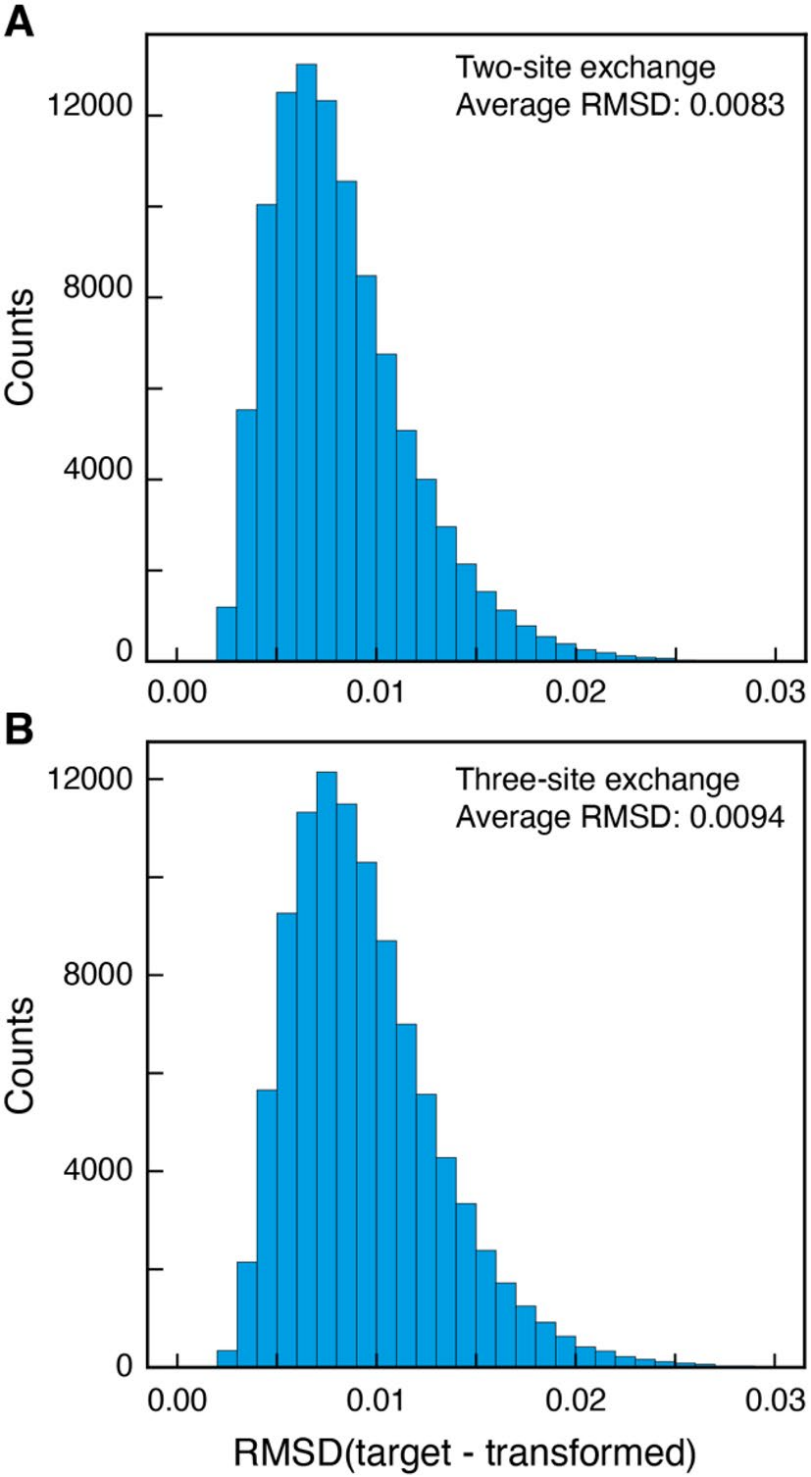
Evaluation of the transformation of anti-phase ^1^H CEST profiles to in-phase CEST profiles using a DNN. **A** RMSD between target and transformed in-phase CEST profile (see Fig. 1). Statistics for 100,000 two-site exchange, G ⇌ E, CEST profiles, where 10 s^−1^ ≤ *k*_ex_ ≤ 300 s^−1^, 0.01 ≤ *p*_E_ ≤ 0.15, 15 Hz ≤ *B*_1_ ≤ 50 Hz, 50 ≤ Sampled points ≤ 128, − 95 Hz ≤ ^1^*J*_HN_ ≤  − 91 Hz, *B*_0_ ∈ {14.1 T, 16.4 T, 18.8 T, 21.1 T, 23.5 T}. **B** Statistics for 100,000 three-site exchange CEST profiles (E_1_ ⇌ G ⇌ E_2_), where 10 s^−1^ ≤ *k*_ex,E1_, *k*_ex,E2_ ≤ 300 s^−1^, 0.01 ≤ *p*_E1_, *p*_E2_ ≤ 0.15, 15 Hz ≤ B_1_ ≤ 50 Hz, 50 ≤ Sampled points ≤ 128, − 95 Hz ≤ ^1^*J*_HN_ ≤  − 91 Hz, *B*_0_ ∈ {14.1 T, 16.4 T, 18.8 T, 21.1 T, 23.5 T}

The trained DNN_TR_ network was evaluated separately on synthetic data for two- and three-site exchanging systems. Figure 2 shows the evaluation on 100,000 randomly generated CEST profiles for two- (Fig. 2A) and three-site (Fig. 2B) exchanging systems. Figure S3 shows the performance of the DNN transformation as a function of the strength of the weak field, *B*_1_, the population of the sparse state E, *p*_E_, the overall exchange rate, *k*_ex_ (*k*_ex_ = *k*_*GE*_ + *k*_*EG*_, for two-site interconversion) and the number of sampled offsets. The transformation of profiles from anti-phase to in-phase by the DNN_TR_ network is robust and there is only limited variation in the performance with different parameters used to generate the CEST profiles. Of particular interest is that the transformation is only minimally affected by the number of points sampled in the input profile, Fig. S3D, suggesting that the upsampling is robust.

**Fig. 3:**
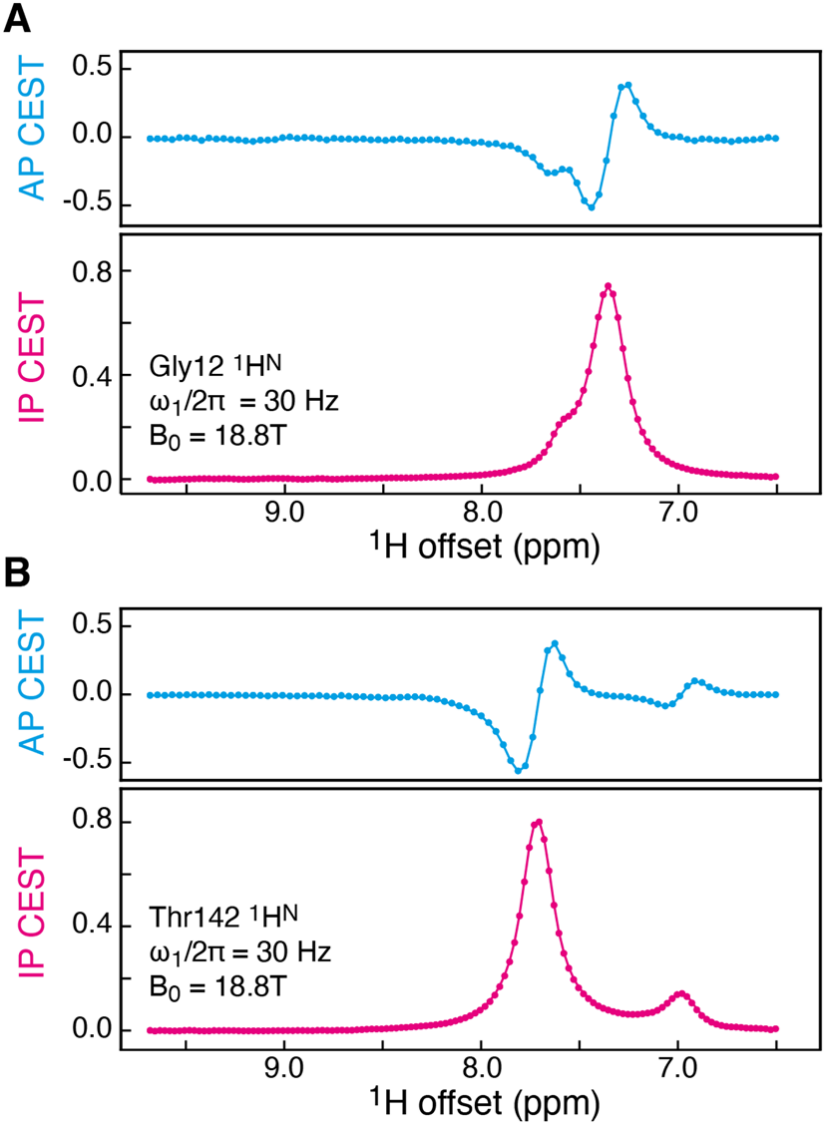
Transformation of experimental anti-phase ^1^H^N^ CEST profiles (AP CEST) recorded on a sample of the L99A mutant of T4 lysozyme into in-phase CEST profiles (IP CEST). The AP CEST profiles were recorded at a static magnetic field of 18.8 T, a temperature of 284 K, and using a 30 Hz ^1^H *B*_1_ field; 86 points were obtained in the CEST dimension. **A** Transformation and upsampling to 128 points of the anti-phase CEST profile for Gly12, **B** transformation and upsampling to 128 points of the anti-phase CEST profile for Thr142

Having evaluated the DNN_TR_ network on synthetic data it is important to assess how the DNN performs on experimental anti-phase ^1^H^N^ CEST profiles. Figure 3 shows two examples, where ^1^H^N^ anti-phase CEST profiles for the L99A mutant of T4 lysozyme recorded at 18.8 T have been transformed to in-phase CEST profiles (with the scalar coupling removed). This representation immediately allows estimation of the chemical shifts of ^1^H nuclei of the exchanging states, which can be used as initial parameters for a least-squares analysis. However, these experimental CEST profiles are associated with uncertainty and since the ground truth (exact value) is not known a detailed evaluation of the performance is not directly possible.

### Determining ^1^H chemical shifts in exchanging states using a deep neural network

With the in-phase CEST profiles available it becomes substantially easier to estimate the chemical shifts of the exchanging species. DNNs are particularly adept at locating specific features in data, for example, localising particular elements in an image. Thus, it is expected that a DNN could be trained to determine the position of peaks in one-dimensional NMR spectra and, consequently, trained to determine the chemical shifts of the exchanging species from in-phase CEST profiles or the related profiles, max(*I*/*I*_0_) − *I*/*I*_0_. The densely connected convolutional neural network architecture (Huang et al. 2016), which was originally developed for object recognition tasks, was adapted here, Fig. S4, to determine the chemical shifts from CEST profiles. Moreover, our goal was not only to determine the chemical shifts of the interconverting conformers, but to also train the DNN to estimate the uncertainties with which it determined these shifts, thereby providing an output similar to a traditional least-squares fitting procedure.

The output from a DNN is typically a fixed length and a decision about the maximum number of exchanging states therefore has to be made before training the network. Since the time for training the DNN increases rapidly when increasing the maximum number of exchanging states, we chose for this application to only focus on CEST profiles reporting on three or less states, which covers most of the CEST-based studies reported to date. For a maximum of three exchanging states the output from the DNN_CS_ network is a 3 × 2 matrix whose elements are three chemical shifts, *f*_*ω,*pred_, and their corresponding confidences, *c*_pred_. When the input CEST profile derives from a two-site exchanging system, the DNN should report one confidence approaching zero and when the input CEST profile is only reporting on one state, two of the confidences should tend to zero.

To facilitate an end-to-end analysis, that is chemical shifts and their uncertainties obtained directly from the experimental anti-phase CEST profiles, the network to determine chemical shifts was trained on outputs from DNN_TR_, i.e. in-phase CEST profiles generated from anti-phase profiles. Having the second DNN, referred to as DNN_CS_, determine both chemical shifts and their confidences requires special attention to the loss function used for training. Naturally, the DNN_CS_ network should be trained to optimise the confidence and thus obtain as accurate peak positions as possible, however, it should also be penalised, when the predicted confidence does not match the accuracy of the predicted chemical shifts. A variety of DNN architectures and loss functions have previously been designed to provide measures of the uncertainty with which DNNs make their predictions and transformations, also for the predictions of chemical shifts (Jonas and Kuhn 2019). As detailed below, we have adopted a strategy, where the loss function bears resemblance with the cost function in a least-squares fitting procedure.

The last layer of DNN_CS_ has sigmoidal activation, Fig. S4, which means that the output values, three values reporting on chemical shifts and three confidences, are between 0 and 1. The predicted chemical shifts in the range (0, 1), referred to as *f*_*ω,*pred_, are easily converted into the range of offsets obtained in the CEST dimension of the original data using a linear mapping. For example, if the CEST profile is recorded with points between 6.6 ppm and 10.0 ppm, then the linear mapping will be *δ* ← 3.4 ppm × *f*_*ω,*pred_ + 6.6 ppm. Moreover, a predicted uncertainty, σ_pred_, was calculated from the predicted confidence as σ_pred_ = *k* (1/*c*_pred_ − 1), where *k* is a constant and σ_pred_ structured such that it can take values between 0 and infinity. In order to make the predicted uncertainties match actual uncertainties of the prediction, the first part of the loss function was defined in a manner similar to a standard *χ*^2^, that is:

1 $$L_{{{\text{freq}}}} = \mathop \sum \limits_{i = 0,1,2} \frac{{\left( {f_{{\omega ,{\text{pred}},i}} - f_{{\omega ,{\text{ true}},i}} } \right)^{2} }}{{\sigma_{{{\text{pred}},i}}^{2} }}$$

where the sum is over the three states. The constant *k* was initially set to 1 during training, and subsequently set to $\left( {\max \left( {{}_{{}}^{1} {\text{H}}\ {\text{offsets}}} \right) - \min \left( {{}_{{}}^{1} {\text{H}}\ {\text{offsets}}} \right)} \right)\sqrt {L_{{{\text{freq}}}} }$ to rescale *L*_freq_ to have an expectation value of 1 and so that σ_pred_ reports on the expected uncertainty. The purpose of the loss function in Eq. (1) is to make the predicted chemical shifts approach their true values. However, if *L*_freq_ was the only loss function used during training, then training of DNN_CS_ would simply lead to very low confidences (high uncertainties), which would minimise the function in Eq. (1). A second loss function was therefore added during training:

2 $$L_{{{\text{uncer}}}} = 10^{ - 4} \mathop \sum \limits_{i = 0,1,2} 1_{i} \sqrt {\sigma_{{{\text{pred}},i}} }$$

where, **1** = {1,1,1} for three-state exchange input and **1** = {1,1,0} in the case of two-state exchange, thereby allowing large uncertainties, σ_pred_, when a state is not present in the input. The loss function in Eq. (2) serves to force DNN_CS_ to predict high confidences (low uncertainties) where, and only where, the input profiles report on a real state. Briefly, the DNN_CS_ network was trained on 1.5 × 10^7^ randomly generated CEST profiles, with a final value of *L*_freq_ = 7.3 × 10^–5^, and *L*_uncer_ = 2.8 × 10^–4^. For the synthetic CEST data analysed below, the range of ^1^H offsets was 3.4 ppm and therefore *k* = 0.029 ppm. Full details of the network architecture and the training are provided in the Methods and Supporting Information sections.

It is anticipated that with minimal additional training, the DNN_CS_ network will be able to accurately analyse common ‘in-phase’ CEST profiles such as those often obtained for ^15^N and ^13^C, since these CEST profiles strongly resemble the IP-CEST profiles, Fig. 3. However, it should be stressed that the current DNN_CS_ network has only been fully assessed with ^1^H AP-CEST profiles that have been transformed with DNN_TR_.

### End-to-end one-shot analysis of amide proton CEST

The two DNNs, DNN_TR_ and DNN_CS_, described above can be applied sequentially to provide an end-to-end one-shot analysis of anti-phase CEST profiles:

$${\text{AP-CEST,}}\,{\mathbf{cest}}_{{{\text{AP}}}} \left( \omega \right){ } \xrightarrow{{{ {\text{real}}\,{\text{FT}},\,{\text{DNN}}_{{{\text{TR}}}} ,\,{\text{inverse}}\,{\text{FT}} }}} {\text{IP-CEST,}}\,{\mathbf{cest}}_{{{\text{IP}}}} \left( \omega \right) \xrightarrow{{{ {\text{DNN}}_{{{\text{CS}}}} }}} \left\{ {f_{{\omega ,\,{\text{pred}},\,i}} ,\,\sigma_{{{\text{pred}},\,i}} } \right\}_{i = 0,\,1,\,2}$$

The overall performance of this sequential DNN was first evaluated using synthetically generated data. Specifically, (i) 100,000 anti-phase CEST profiles were generated for a variety of two-site chemical exchange processes and a further 100,000 profiles for three-site exchange. The range of B_1_ offsets used was 3.4 ppm for all profiles, and all other input parameters are given in Table 1. (ii) Random gaussian noise with a standard deviation of 0.01 of the maximum value of each anti-phase CEST profile was added to the input anti-phase CEST spectrum. (iii) The DNN_TR_ network was first used to transform all the CEST profiles from anti-phase to in-phase. (iv) The second network, DNN_CS_, was used to determine the chemical shifts of the exchanging states and their associated uncertainties.

**Fig. 4:**
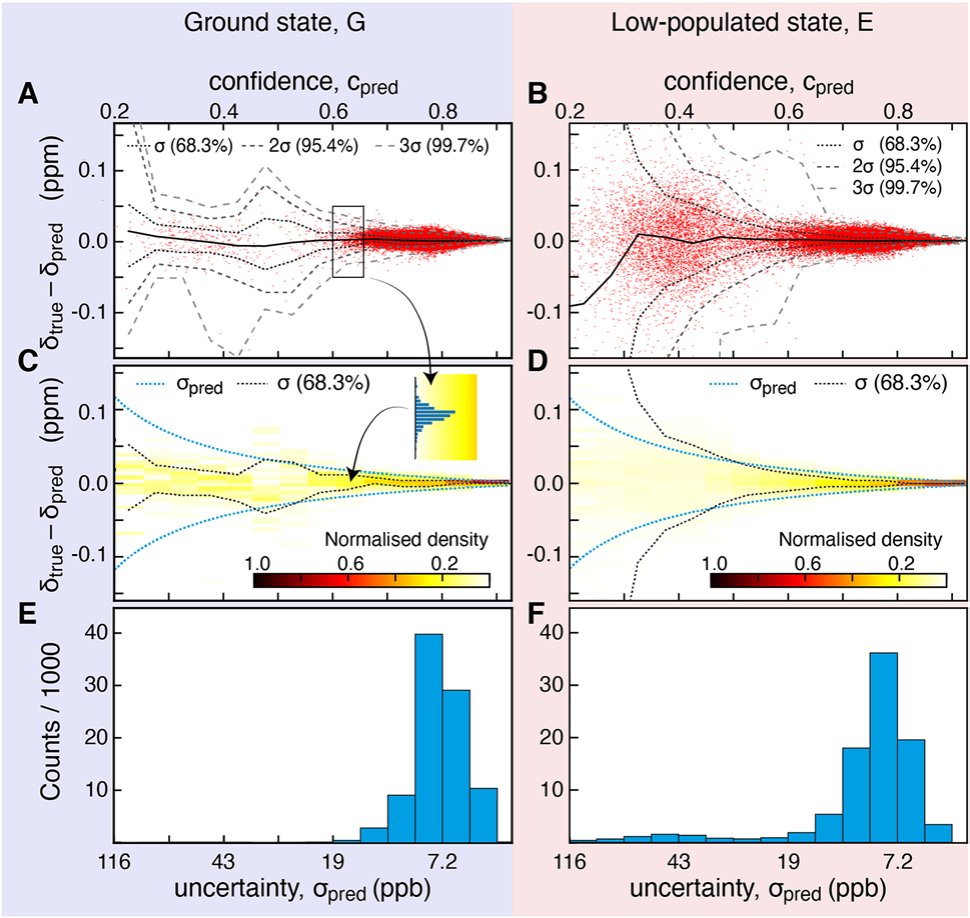
Quantitative assessment using 100,000 synthetic anti-phase ^1^H^N^ CEST profiles reporting on a two-site chemical exchange and analysed using the sequential DNN to determine the chemical shifts of nuclei from the exchanging states. **A**, **C** and **E** assessment of the ground-state predictions, **B**, **D** and **F** assessment of the predictions of the low-populated state. **A** and **B** show differences between predicted (*δ*_pred_) and true (*δ*_true_) chemical shift values, *versus c*_pred_ for the 100,000 analysed CEST profiles (red dots). The full-drawn line corresponds to the average and the dashed lines correspond to the standard confidence levels, 68.3%, 95.4%, and 99.7%, respectively. **C** and **D** show 2D histograms of the points in (**A**) and (**B**); that is, a 2D histogram of the differences between *δ*_pred_ and *δ*_true_ *versus* the predicted confidence, *c*_pred_. The histogram was calculated with a resolution of 0.05 along *c*_pred_ and 0.005 ppm along *δ*_true_ − *δ*_pred_. The blue dashed lines show the predicted uncertainty, σ_pred_ = 0.029(1/*c*_pred_ − 1), which for *c*_pred_ > 0.4 agrees well with the confidence levels obtained from the analysis of the 100,000 profiles. **E** and **F** shows the distributions of uncertainties obtained from the assessment

Figure 4 shows a summary of the quantitative assessment of the 100,000 CEST profiles corresponding to a two-state chemical exchange process. From Fig. 4 it is clear that the sequential DNN is able to accurately predict the chemical shifts of exchanging states from anti-phase CEST profiles. From the chemical shift predictions made on the 100,000 random CEST profiles the difference between a predicted chemical shift, *δ*_pred_, and a true chemical shift, *δ*_true_, was calculated, which gives an estimate of the performance and the confidence levels of the DNN as a function of *c*_pred_ and σ_pred_. Importantly, as shown in Fig. 4C and D, the DNN has also successfully been trained to predict the uncertainty associated with the predicted chemical shifts. Specifically, for *c*_pred_ ≥ 0.4, the predicted uncertainty, σ_pred_, agrees well with the 68.3% confidence level estimated from the analysis of the 100,000 profiles. For *c*_pred_ < 0.4, σ_pred_ is no longer an accurate measure of the uncertainty. Not surprisingly, the ground state chemical shifts, Fig. 4E, are generally predicted with a higher accuracy than the chemical shifts of the low-populated state, Fig. 4F, where lower confidences are obtained for small chemical shift differences between the two states, see Fig S5. The corresponding assessment carried out on 100,000 synthetic anti-phase ^1^H^N^ CEST profiles reporting on a three-site chemical exchange process, E_1_ ⇌ G ⇌ E_2_, is shown in Supporting Material, Fig. S6. Figure S7 shows the summary of evaluations where random gaussian noise with a standard deviation of 0.01, 0.02, 0.04 of the maximum value of each anti-phase CEST profile was added to the input anti-phase CEST spectrum. The performance of the sequential DNN shown above strictly only holds for the ranges of data that were used for training and for the quantitative assessments, Table 1. However, as shown below, the performance of the DNN is rather robust and if the parameters of the CEST profile to be analysed deviate only slightly from the training parameters one would still expect the analysis to be valid. The ranges of parameters shown in Table 1 cover those obtained in most of CEST-based studies to date and it is therefore expected that most experimental anti-phase CEST profiles can be accurately analysed using the DNNs.

### Assessment of the sequential DNN to analyse experimental CEST profiles

**Fig. 5:**
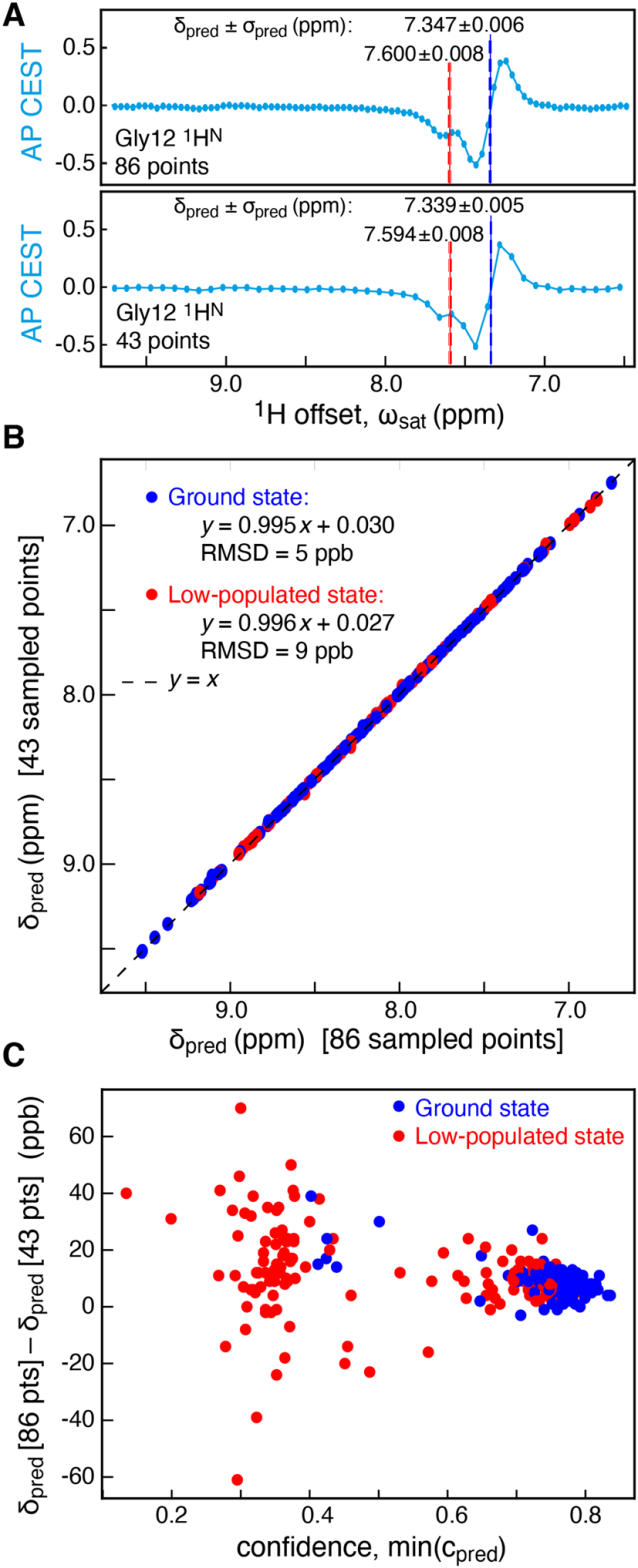
Predicting the chemical shifts of exchanging states of L99A T4 Lysozyme. The DNN for the transformation of anti-phase to in-phase profiles, DNN_TR_, upsampled the recorded data to 128 points and DNN_CS_ determined the chemical shifts. Two full analyses were performed: One on the original 86 points recorded and another analysis on half of the data. **A** Analysis of the anti-phase profile for Gly12 ^1^H^N^ emphasizes the robustness by which the sequential DNN determines chemical shifts and their predicted uncertainties. **B** Consistency plot showing excellent agreement between the chemical shifts determined from the full dataset (*x*-axis) and half of the data (*y*-axis). Only data for which *c*_pred_ > 0.4 are shown. **C** Differences between the predicted chemical shifts from the full dataset (*δ*_pred_ [86 pts]) and half of the data (*δ*_pred_ [43 pts]) versus the minimum of the confidence, min(*c*_pred_) = min(*c*_pred_ [86 pts], *c*_pred_ [43 pts]). All data are included

Experimental anti-phase ^1^H CEST profiles for the L99A mutant of T4 lysozyme were analysed using the sequential and stacked DNN to gain insight into its performance on experimental data. As a validation of the performance of the fully stacked DNN two analyses were performed: in the first all of the 86 *B*_1_ offsets were used to predict chemical shifts, while in the second, half of the offsets (every second point) were removed. Figure 5A shows the example of Gly12, where the predicted chemical shifts and uncertainties using half of the *B*_1_ offsets agrees well with the values obtained using the full dataset. Generally, this holds for all sites, Fig. 5B and the RMSDs obtained are in line with those expected from the predicted uncertainties, σ_pred_. The differences in chemical shifts based on analyses of the full and half datasets, for all profiles, as a function of the confidence level are highlighted in Fig. 5C. Finally, it should be noted that the DNN_TR_ network was only trained on profiles with 50–128 input points. The fact that the stacked DNN is able to accurately predict the chemical shifts from profiles with less data (43 points) than those used for training points to the robustness of the DNN.

**Fig. 6:**
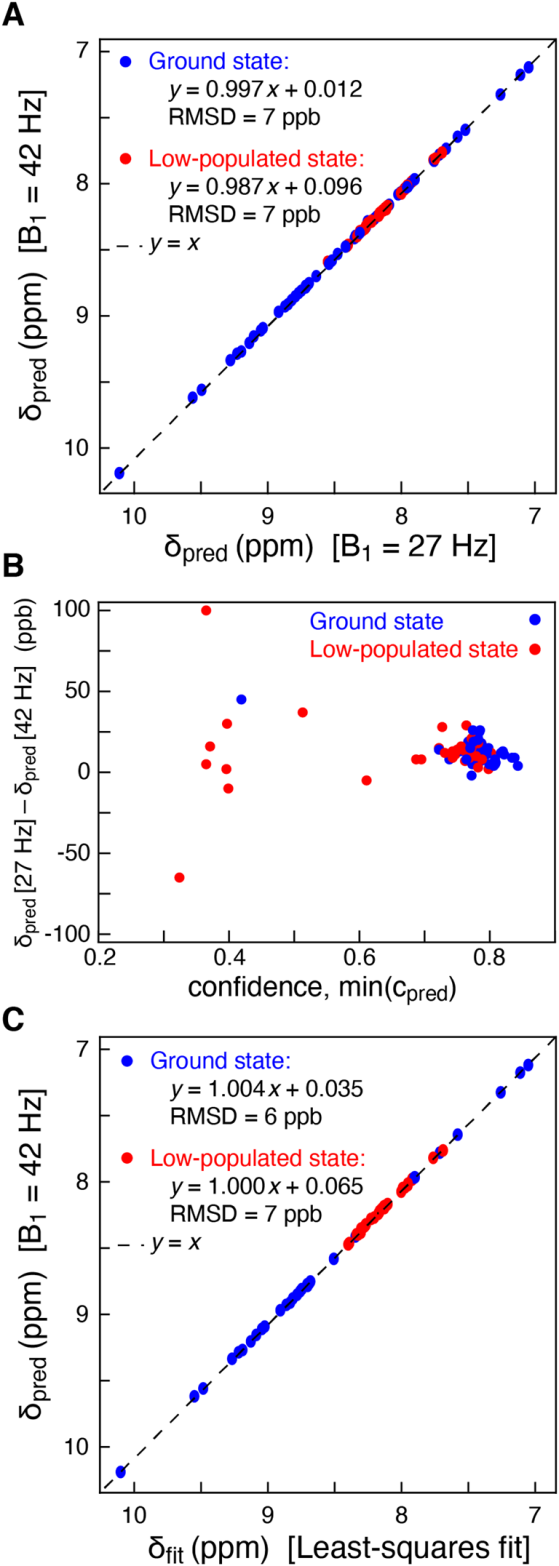
Predicting the chemical shifts of exchanging sites of the G48A mutant of the SH3 domain from Fyn. **A** Two datasets were recorded using different *B*_1_ field strengths, 26.7 Hz and 42 Hz. The consistency plot shows that the chemical shifts determined independently from the two datasets agree. **B** Differences between the predicted chemical shifts from the two datasets (*δ*_pred_ [27 Hz] and *δ*_pred_ [42 Hz]) versus the minimum of the confidence, min(*c*_pred_) = min(*c*_pred_ [27 Hz], *c*_pred_ [42 Hz]). All data are included. **C** Consistency plot showing that the chemical shifts determined using the stacked DNNs (one dataset) agree with the chemical shifts determined from least-squares fitting (two datasets). Only data for which *c*_pred_ > 0.4 are shown

To further assess the performance of the stacked DNNs in determining the chemical shifts of the exchanging states, anti-phase CEST profiles were obtained for the G48A mutant of the SH3 domain from Fyn (Yuwen et al. 2017a). At a static magnetic field of 14.1 T, two sets of data were obtained with *B*_1_ fields of 26.7 Hz and 42 Hz. Figure 6A shows that the chemical shifts predicted using the stacked DNNs, independently, on the two different datasets agree well (RMSD of 7 ppb), and Fig. 6B highlights the difference in shifts based on the separate analyses of the two full datasets. Subsequently, the two experimental datasets were analysed simultaneously using a standard least-squares analysis (Yuwen et al. 2017a) with the software package ChemEx (https://github.com/gbouvignies/chemex) and the results were compared with the predictions made by the DNN, Fig. 6C. Again, the agreement between the chemical shifts predicted by the DNN and those obtained by least-squares fitting agree well, with an RMSD of 7 ppb.

Uncertainties obtained from the covariance matrix in a least-squares analysis of CEST profiles are typically around 1 ppb, which is 6 times smaller than the uncertainties obtained from the DNN, indicating that the stacked DNNs have not fully reached the level of accuracy obtained by least-squares fitting. Still, the predictions obtained from the analysis with the stacked DNNs are of an accuracy where they can be used for downstream analyses and are well beyond the level of accuracy by which these shifts can be predicted from a high-resolution structure (Han et al. 2011). Alternatively, the DNN-predicted chemical shifts can serve as excellent starting parameters for a subsequent least-squares analysis. It is also possible that larger or alternative DNN architectures along with longer training periods could improve the performance of the DNN predictions.

## Conclusions

A deep neural network was developed and trained to determine amide proton chemical shifts of exchanging states from anti-phase ^1^H^N^ CEST profiles. The approach first leads to the conversion of anti-phase to in-phase ^1^H^N^ CEST profiles, whereafter the chemical shifts are predicted along with their uncertainties. Compared with other analysis tools, the DNN does not require any additional training and there are no user adjustable parameters, which makes the analysis autonomous and suitable for automated processing pipelines. Thus far, the DNN only predicts chemical shifts. If additional parameters are sought, such as exchange rates and populations, the output shift values from the DNN can then serve as excellent starting points for a least-squares fitting procedure. The methodology and DNNs presented here add to the growing applications of deep learning and artificial intelligence for the analysis of NMR data, and provide an example of the autonomous analysis of complex NMR data reporting on macromolecular dynamics and chemical exchange.

## Supplementary Information

Below is the link to the electronic supplementary material.

Supplementary file1 (PDF 1942 KB)

## Data Availability

The datasets generated during and/or analysed during the current study are available from the corresponding author on reasonable request. The training data are also available as open source from Zenodo (Karunanithy et al. 2022) with scripts to analyse these. Moreover, scripts and code for performing the end-to-end one-shot analysis of amide proton CEST on experimental data using DNN_TR_ and DNN_CS_ are available on GitHub: https://github.com/gogulan-k/DNN_autoCEST.
