# Supplementary material for: Towards autonomous analysis of chemical exchange saturation transfer experiments using deep neural networks
Source: J Biomol NMR. 2022 May 27;76(3):75–86. doi: 10.1007/s10858-022-00395-z (PMC9246985; doi:10.1007/s10858-022-00395-z)
Supplement: Supplementary file 1 — Supplementary file1 (PDF 1942 KB) [file 10858_2022_395_MOESM1_ESM.pdf]

Supporting Material for

Towards autonomous analysis of Chemical Exchange Saturation Transfer experiments using Deep Neural Networks

Gogulan Karunanithy, Tairan Yuwen, Lewis E Kay, and D Flemming Hansen

The real Fourier transform applied to anti-phase CEST

A real Fourier transform is used in this study to transform the original frequency-domain anti-phase CEST profiles to time-domain signals, each resembling an FID; this facilitates 'decoupling' with a DNN architecture similar to our previous FID-Net[1]. When applying a discrete Fourier transform (DFT) to frequency domain NMR spectra one typically applies the DFT to complex data (real, imaginary). The anti-phase CEST profiles consist of pure real values without an imaginary component, and we therefore use a real Fourier transform to generate the time-domain signal, without the redundancy that would accompany a complex DFT (see below).

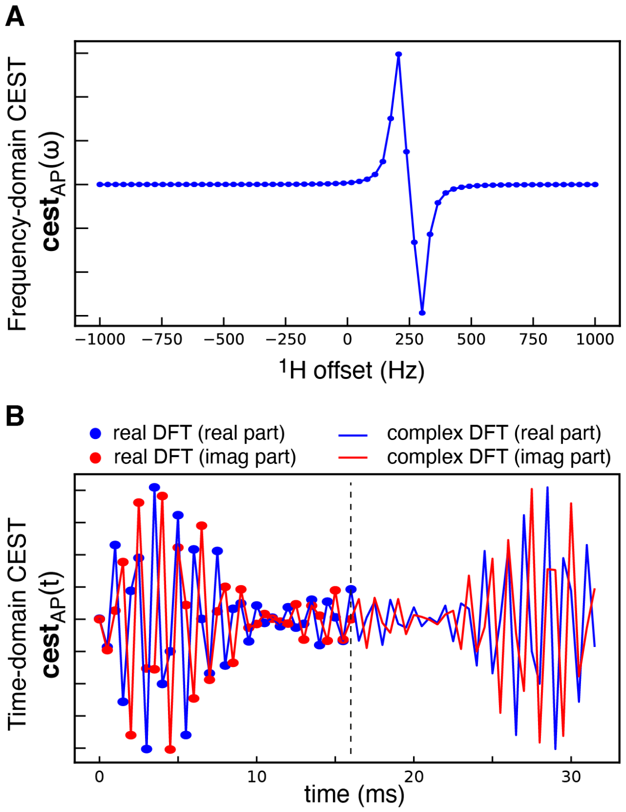

Fig. S1. (A) A synthetic frequency domain $^{1}H^{N}$ anti-phase CEST profile. The profile has been sampled with 64 B_1 offsets (points), the $^{1}J_{HN}$ scalar coupling is –92 Hz, and the resonance frequency is 250 Hz. (B) Fourier transforms of the frequency domain $^{1}H^{N}$ anti-phase CEST profile. The classical complex discrete Fourier transform (complex DFT) of the frequency domain anti-phase CEST profile is shown with continuous lines, while the real discrete Fourier transform is shown with filled circles. The time-domain is calculated from the range of ^{1}H offsets in the CEST profile (x-axis; 2000 Hz), with t = {0,1,…,63}/(2000 Hz).

To illustrate this, the synthetic anti-phase CEST profile in Fig S1A is considered. For a discrete complex Fourier transform of pure real data (N = 64 data points), Fig S1B, the

output is Hermitian-symmetric so that approximately half ($N/2-1$ for even N and $(N-1)/2$ for odd N) of the points are therefore redundant. This is also clear from Fig S1, where the last 31 complex points form a Hermitian-symmetric reflection about point $N/2+1$ (the 33rd point; dashed line in Fig S1B). Thus, points $N/2+1+i$ and $N/2+1-i$ are complex conjugate pairs. A real Fourier transform only retains the $N/2+1$ non-redundant points and a real FT is, therefore, used to transform the frequency-domain CEST profiles to avoid redundant input to the DNN.

Supporting Figures

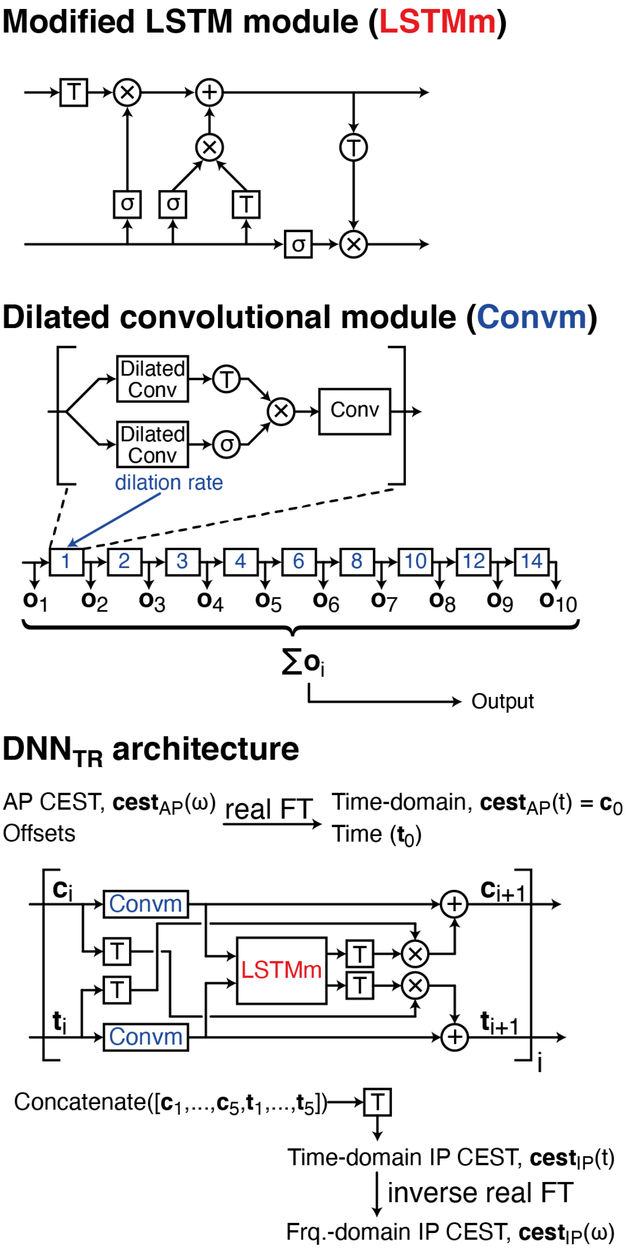

Fig. S2. Neural network architecture for the DNN used to transform anti-phase CEST profiles (AP CEST) to in-phase CEST profiles, consisting of a modified LSTM module (LSTMm) and a convolutional module (Convm). For the architecture used here, $i = \{0, 1, 2, 3, 4\}$. A box represents a layer with trainable parameters, that is, a tensor multiplication followed by an activation, whereas a circle represents an elementwise operation. A box with ‘T’ denotes a linear layer with $\{\tanh(x) + 0.02x\}$ activation, ‘σ’ denotes a linear layer with $\{\mathrm{sigmoid}(x) + 0.02x\}$ activation. The $\tanh(x) + 0.02x$ and $\mathrm{sigmoid}(x) + 0.02x$ activation functions were used instead of $\tanh(x)$ and $\mathrm{sigmoid}(x)$ to obtain better training of the DNN. A circle with ‘+’ denotes an elementwise addition layer, and a circle with ‘×’ denotes an elementwise multiplication layer. All details are provided in Table S1 and in the source code.

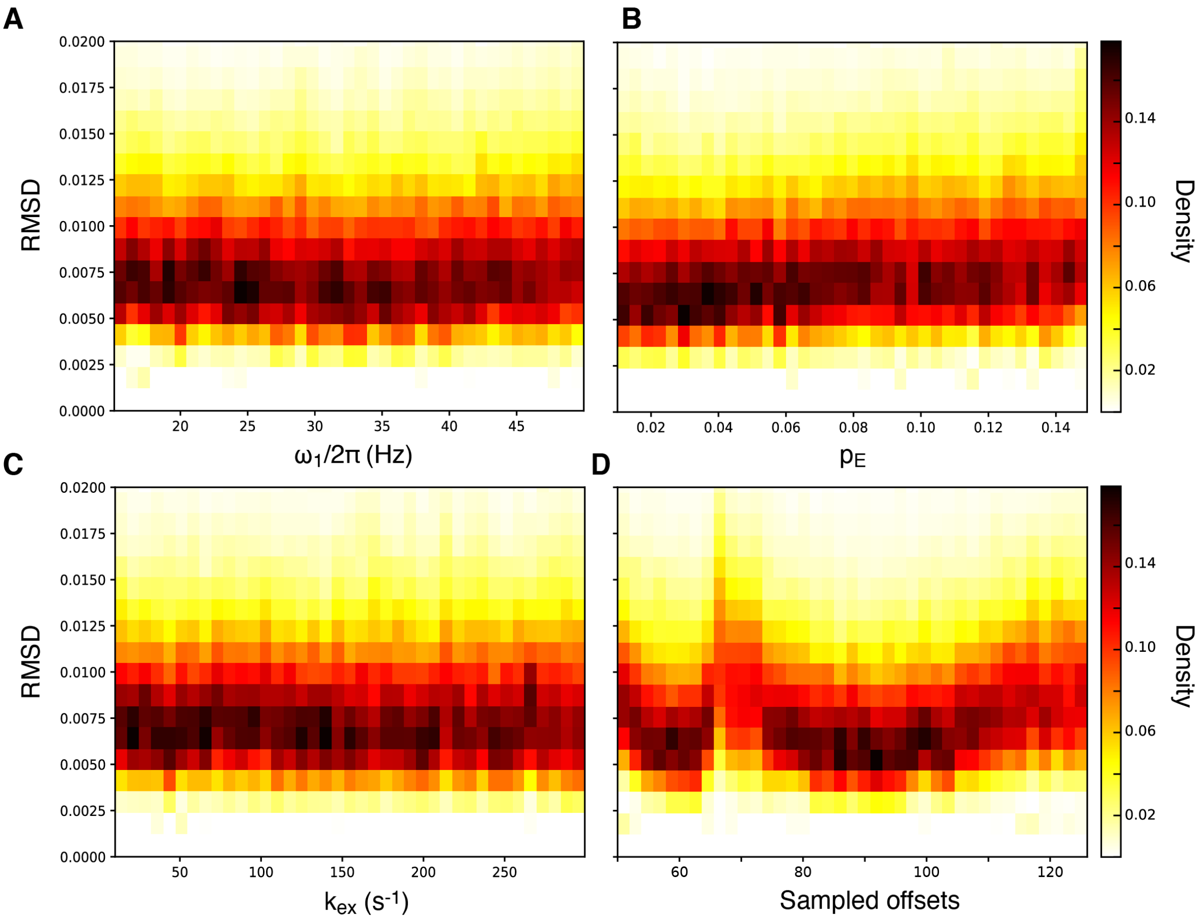

Fig. S3. Evaluation of the DNN_{TR} network, which transforms anti-phase to in-phase CEST profiles for a two-site chemically exchanging system, G ⇌ E, considering the applied B_1 field, the chemical exchange parameters, and the number of sampled points in the CEST frequency dimension. Shown are two-dimensional histograms depicting the probability of a given root-mean-square deviation (RMSD) *versus* a given parameter from a set of parameters used to generate the CEST profiles. The RMSD is defined as $\sqrt{\text{mean}\left\{\left(\text{DNN}_{\text{TR}}(\mathbf{CEST}_{\text{AP}}(\omega)) - \mathbf{CEST}_{\text{IP,target}}(\omega)\right)^2\right\}} = \sqrt{\text{mean}\left\{\left(\mathbf{CEST}_{\text{IP,pred}}(\omega) - \mathbf{CEST}_{\text{IP,target}}(\omega)\right)^2\right\}}$ (A) RMSD *versus* the field strength of the applied, B_1 field. (B) RMSD *versus* the population of the low populated state, E. (C) RMSD versus the overall exchange rate, k_{ex}. (D) RMSD *versus* the number of points sampled in the input anti-phase CEST profile.

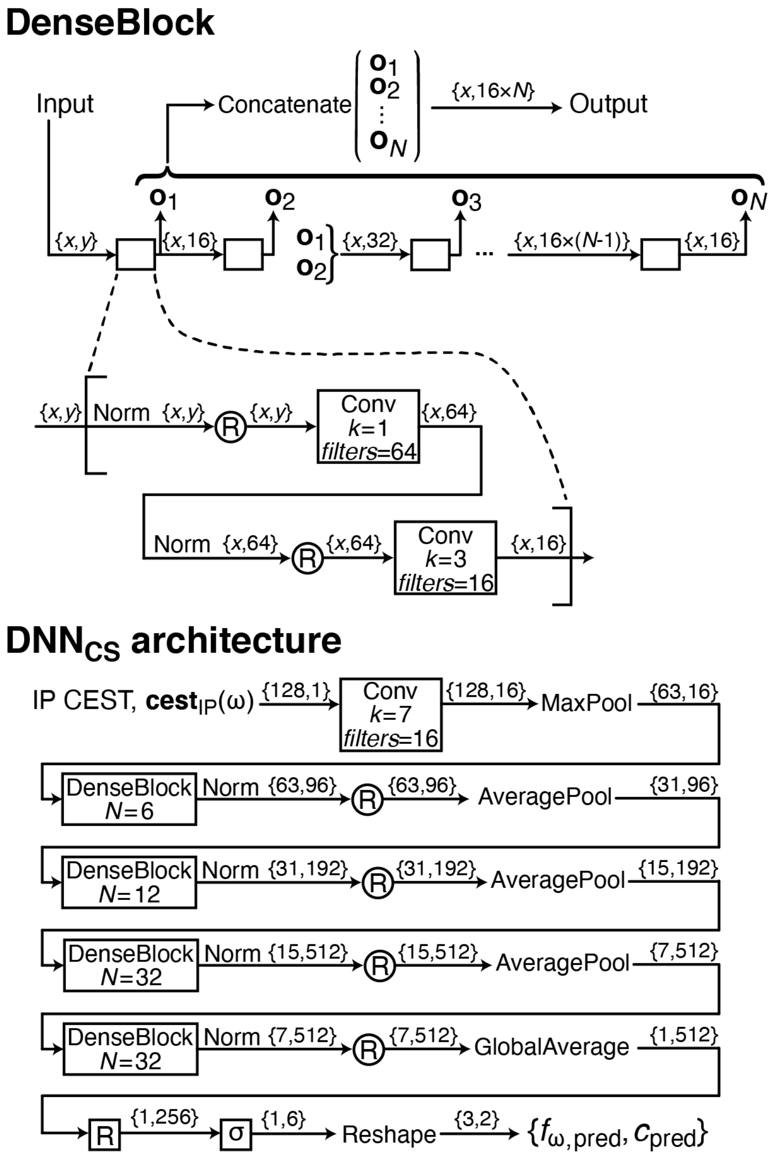

Fig. S4. Schematic representation of the DNN(cs) neural network architecture used to determine chemical shifts from the frequency-domain in-phase CEST profiles. A box represents a layer with trainable parameters, that is, a tensor multiplication followed by an activation, whereas a circle represents an elementwise operation. A box with 'Conv, k=kernel_size' refers to a one-dimensional convolutional layer with kernel size kernel_size and a dilation rate of 1 (no dilation). The numbers above the arrows show the size of the tensor being transferred. The operation, 'Norm' refers to a batch normalisation layer, 'MaxPool' refers to a one-dimensional MaxPool layer, and 'AveragePool' refers to an average pooling layer. A box with 'σ' denotes a linear layer with sigmoid(x) activation and a circle with 'σ' refers to elementwise sigmoidal activation. Similarly, 'R' denotes a rectified linear unit, $x \rightarrow (x + |x|)/2$. The predictions follow a sigmoidal activation and $f_{\omega,\text{pred}}$ and c_{pred} are therefore between 0 and 1. All details are provided in Table S1 and in the source code.

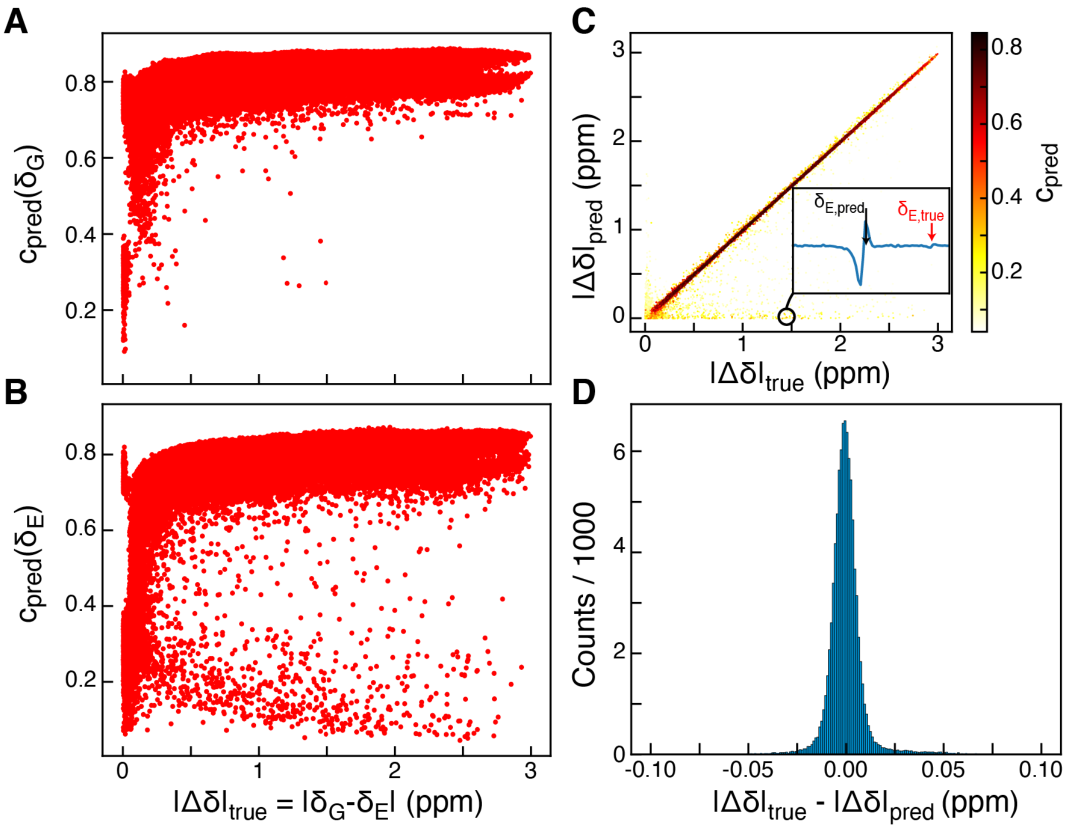

Fig S5. Assessment of the predicted confidence, c_{pred}, for two-site chemically exchanging systems, G $\rightleftharpoons$ E, where random Gaussian noise was added with a standard deviation of 0.01 of the maximum value of each anti-phase CEST profile. (A, B) For 100,000 random anti-phase CEST profiles the predicted confidence of chemical shifts of nuclei from either ground (A) or low-populated (B) states has been plotted against the difference in chemical shifts between the ground-state, δ_{G}, and the low-populated state, δ_{E}, $|\Delta\delta|$. (A) shows the predicted confidence for δ_{G}, whereas (B) shows the confidence for δ_{E}. A clear trend is observed, where low confidence (large uncertainty) is mainly observed for small chemical shifts differences, $\Delta\delta$. For small $\Delta\delta$ the DNN occasionally predicts the chemical shift of the ground state with lower confidence than the low-populated state. This is merely a reflection of the fact that only one chemical shift can be confidently predicted. (C) Predicted chemical shift differences, $|\Delta\delta|_{\text{pred}} = |\delta_{\text{G,pred}} - \delta_{\text{E,pred}}|$ plotted against the true chemical shift differences, $|\Delta\delta|_{\text{true}} = |\delta_{\text{G,true}} - \delta_{\text{E,true}}|$. The 100,000 points in the scatter plot have been coloured according to the confidence, $c_{\text{pred}} = \min(c_{\text{pred}}(\delta_{\text{E}}),\ c_{\text{pred}}(\delta_{\text{G}}))$. For the small fraction of points with $|\Delta\delta|_{\text{pred}} \lesssim 0.1$ and $|\Delta\delta|_{\text{true}}$ up to approximately 1.5 ppm, the anti-phase CEST profiles typically have small ‘dips’ close to the noise for the low-populated state, which is generally caused by small k_{ex} and/or small p_{E}. One such example is shown in the inset, where B_1 = 32 Hz, k_{ex} = 16 s^{-1}, and p_{E} = 2.4%. (D) Histogram showing the distribution of differences between true chemical shift differences, $|\Delta\delta|_{\text{true}}$, and predicted chemical shift differences, $|\Delta\delta|_{\text{pred}}$.

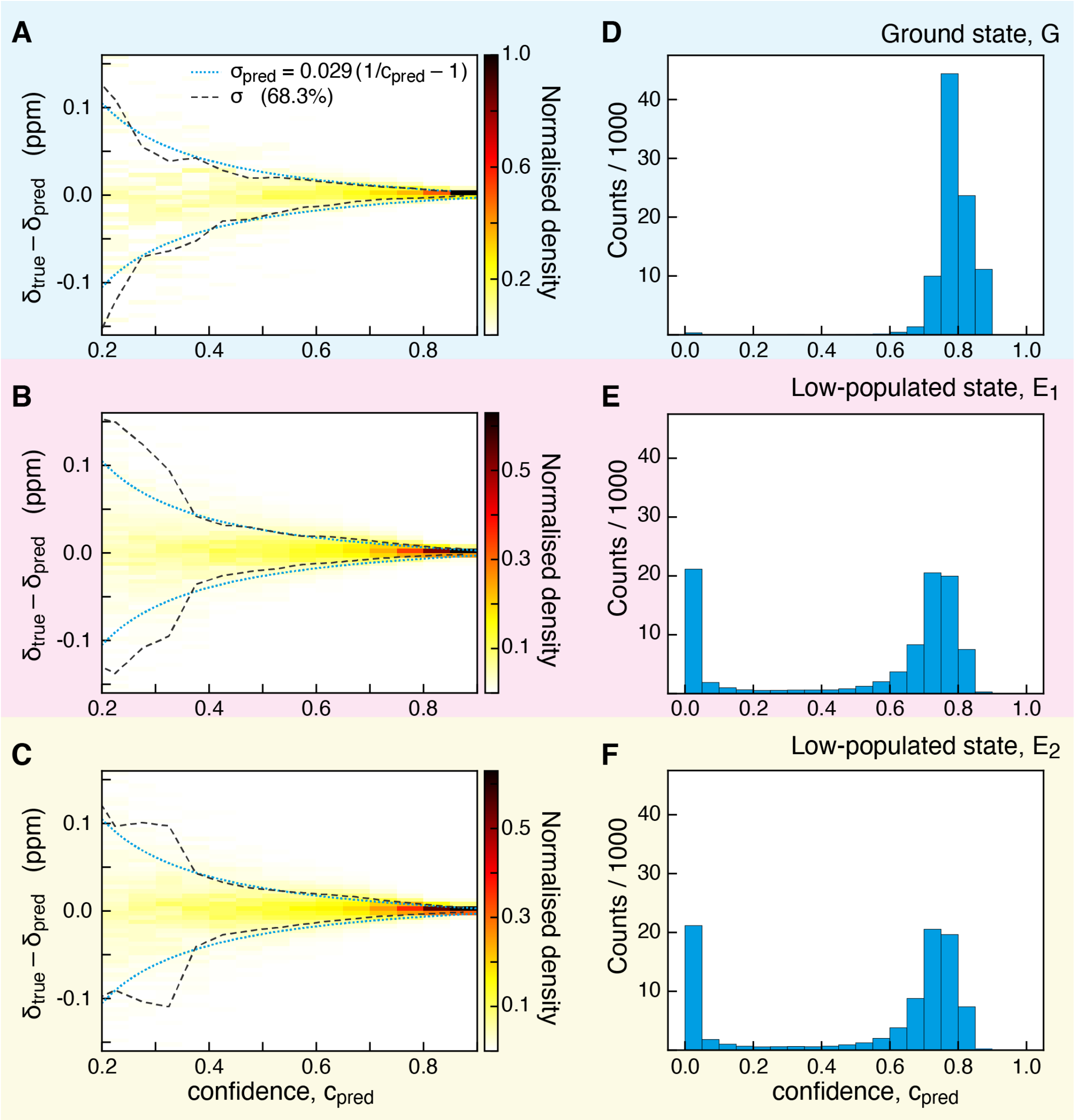

Fig S6. Quantitative assessment of sequential DNNs for the determination of chemical shifts from anti-phase $^1H^N$ CEST profiles. 100,000 synthetic anti-phase $^1H^N$ CEST profiles reporting on a three-site chemical exchange process, $E_1 \rightleftharpoons G \rightleftharpoons E_2$, were generated, analysed via the sequential DNN procedure described in the text, and the accuracy of predicted chemical shifts for ground (A,D), and low-populated (E_1: B,E; E_2: C, F) states assessed. (A, B, C) show two-dimensional histograms for the 100,000 CEST profiles of the difference between predicted δ_{pred}, and true chemical shifts, δ_{true}. The histogram is calculated with a c_{pred} interval of 0.05 (see Fig 4 for details). An overlay is made with the 68.3% confidence level for the analysed CEST profiles as a function of c_{pred} (black dashed line), as well as the predicted uncertainty, σ_{pred}. For $c_{pred} > 0.4$, the predicted uncertainty, σ_{pred}, agrees well with the confidence levels obtained from the analysis of the 100,000 profiles. (D,E,F) show the distributions of confidences obtained from the assessment. In cases where c_{pred} values are close to 0 only a single dip is observed in CEST profiles.

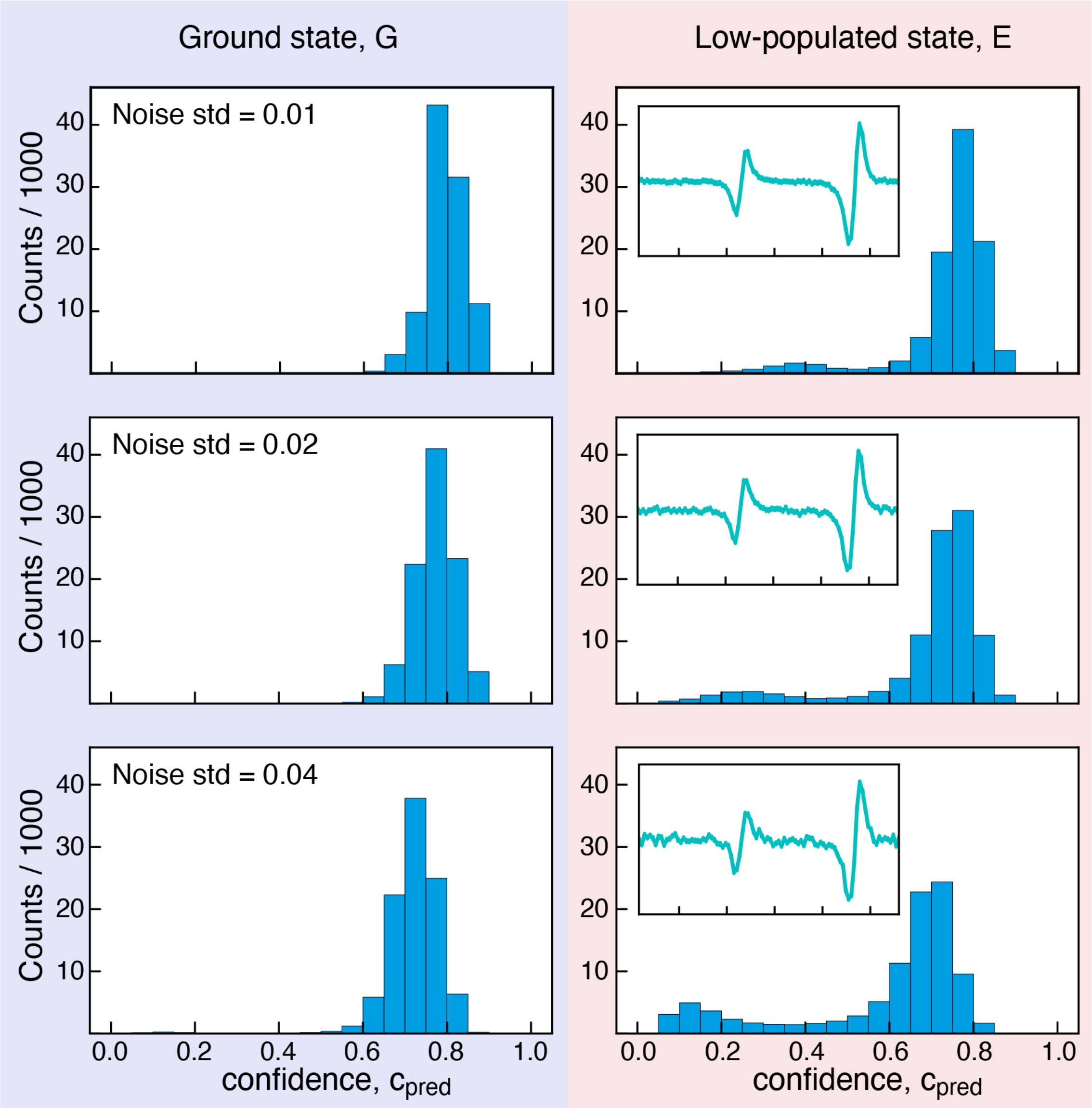

Fig S7. Quantitative assessment of sequential DNNs with regards to the distribution of the expected confidence, c_{pred}, when increased Gaussian noise is added to the input anti-phase CEST profiles. The 100,000 synthetic anti-phase $^{1}H^{N}$ CEST profiles reporting on two-state chemical exchange were generated as described in the main text. The top row represents the assessment presented in Fig 4, where Gaussian noise with a standard deviation of 0.01 was added to the input anti-phase CEST profiles. The anti-phase CEST profile shown as an inset in the right column is the same profile as that shown in Fig 1A. In the second row, Gaussian noise with a standard deviation of 0.02 was added to the input profiles, and in the bottom row Gaussian noise with a standard deviation of 0.04 was added. Histograms in the left column report on the predicted confidences for the ground-state chemical shifts, and the histograms in the right column report on the confidences for the predicted chemical shifts of the low-populated state, E.

Table S1: Description of the layers used in the Neural Networks DNN_{TR} and DNN_{CS}:

Layer symbol	Operations
T (boxed)	A linear layer with hyperbolic tangent, tanh(x), activation function and bias. The layer transforms, in a linear manner, an input vector **x** into an output vector **y**. This layer contains a parameter-tensor, **A**, and a parameter-vector, **b**. Specifically, **y** = {tanh(z_1), tanh(z_2), …, tanh(z_n)}, where **z** = {z_1, z_2, …, z_n} and **z** = **Ax** + **b**. Training the neural network involves optimisation of all the parameters of **A** and **b**.
T (circled)	An elementwise operation, $x \rightarrow$ tanh(x). The layer transforms an input vector **x** into an output vector **y**, specifically, **y** = {tanh(x_1), tanh(x_2), …, tanh(x_n)}. There are no parameters to be optimised.
σ (boxed)	A linear layer with sigmoidal activation and bias. This layer is similar to the linear 'T' layer described above, only difference is that a sigmoidal function is used, $\varphi(x) = (1+\exp(-x))^{-1}$, instead of the hyperbolic tangent.
σ (circled)	An elementwise operation, $x \rightarrow \varphi(x)$, where $\varphi(x) = (1+\exp(-x))^{-1}$. The layer transforms an input vector **x** into an output vector **y**, specifically, **y** = { φ(x_1), φ(x_2), …, φ(x_n)}. There are no parameters to be optimised.
Conv (boxed)	A one-dimensional convolutional layer, which contains a parameter-kernel, **k** = {k_1, k_2, … } of size *kernel_size* and a bias *b*. For an input vector **x** of size *n*, the input vector is first zero-filled with *kernel_size* – 1 zeros. Either (*i,* DNN_{TR}) all zeros added to the end of the input or (*ii*, DNN_{CS}) zeros will be added to both the beginning and end of the input vector and if an odd number of zeros are required one additional zero is added to the end. After zero-filling, the output vector, **y**, is given by **y** = $\{b + \sum k_i x_i$, $b + \sum k_i x_{i+1}$, … }. Training the neural network involves optimisation of all the parameters of **k** and *b*.<br>As an example, consider the following vector **x** = {0.5, 0.5, 0.9, 2.0, 0.5, 2.0, 0.9, 0.5, 0.5}, whose elements when plotted as intensities give a 'low resolution' doublet, flanked by baseline. For a kernel **k** = {0, 1, 1} and a bias *b*= -1 then the output **y** will be calculated by first adding kernel_size – 1 = 2 zeros to {0.5, 0.5, 0.9, 2.0, 0.5, 2.0, 0.9, 0.5, 0.5, 0.0, 0.0} → **x**. The convolution is now given by:<br>**y** = { 0*0.5 + 1*0.5 + 1*0.9 – 1,<br>0*0.5 + 1*0.9 + 1*2.0 – 1,<br>0*0.9 + 1*2.0 + 1*0.5 – 1,<br>0*2.0 + 1*0.5 + 1*2.0 – 1,<br>0*0.5 + 1*2.0 + 1*0.9 – 1,<br>0*2.0 + 1*0.9 + 1*0.5 – 1,<br>0*0.9 + 1*0.5 + 1*0.5 – 1,<br>0*0.5 + 1*0.5 + 1*0.0 – 1,<br>0*0.5 + 1*0.0 + 1*0.0 – 1} = {0.4, 1.9, 1.5, 1.5, 1.9, 0.4, 0, -0.5, -1}.

Dilated Conv

A one-dimensional dilated convolutional layer, which contains a parameter-kernel, $\mathbf{k} = \{k_1, k_2, \dots\}$ of size *kernel_size* and a bias b. The dilation rate is d. For an input vector $\mathbf{x}$ of size n, the input vector is first zero-filled with $d\times(kernel\_size - 1)$ zeros. Subsequently the output vector, $\mathbf{y}$, is given by $\mathbf{y} = \{b + \sum k_i x_{1+d\times(i-1)}, b + \sum k_i x_{2+d\times(i-1)}, \dots\}$. Training the neural network involves optimisation of all the parameters of $\mathbf{k}$ and b.

As an example, consider the example above with $\mathbf{x}$ = {0.5, 0.5, 0.9, 2.0, 0.5, 2.0, 0.9, 0.5, 0.5}. For a dilation rate = 2, a kernel $\mathbf{k}$ = {0, 1, 1}, a bias b = -1, then the output $\mathbf{y}$ will be calculated by first adding 2*(3 – 1) = 4 zeros to give {0.5, 0.5, 0.9, 2.0, 0.5, 2.0, 0.9, 0.5, 0.5, 0.0, 0.0, 0.0, 0.0} → $\mathbf{x}$. The convolution is now given by:

y = { 0*0.5 + 1*0.9 + 1*0.5 – 1,
0*0.5 + 1*2.0 + 1*2.0 – 1,
0*0.9 + 1*0.5 + 1*0.9 – 1,
0*2.0 + 1*2.0 + 1*0.5 – 1,
0*0.5 + 1*0.9 + 1*0.5 – 1,
0*2.0 + 1*0.5 + 1*0.0 – 1,
0*0.9 + 1*0.5 + 1*0.0 – 1,
0*0.5 + 1*0.0 + 1*0.0 – 1,
0*0.5 + 1*0.0 + 1*0.0 – 1}= {0.4, 3, 0.4, 1.5, 0.4, -0.5, -0.5, -1, -1}

The convolutional layers used for DNN_{TR} and DNN_{CS} have several output 'filters', meaning that the convolutional layer effectively contains multiple kernels of the same size (and one b per kernel), thereby generating multiple output vectors $\mathbf{y}$. More specifically, consider an input, $\mathbf{x}$, consisting of I input filters and a length L. This input, $\mathbf{x}$, can be described by a matrix of size {L,I} and its elements denoted $\mathbf{x}_{l,i}$. Now consider the kernel $\mathbf{k}$, with a kernel size *kernel_size*. If we have J output filters, then the total kernel used for the one-dimensional convolution can be described with a tensor of size {*kernel_size*,I,J}. After zero-filling of the input with *kernel_size* -1 zeros, and with a dilation rate of 1 (so that the input $\mathbf{x}$ consists of L+kernel_size-1 elements), the output of size {L,J} is given by $\mathbf{y}_{l,j} = \sum_i \sum_k \mathbf{x}_{k+l-1,i} \times \mathbf{k}_{k,i,j}$.

R

A linear layer with rectified linear activation and bias. This layer is similar to the linear 'T' layer described above, only difference is that a rectified linear unit function is used, $\varphi(x) = (x + |x|)/2$, instead of the hyperbolic tangent.

(R)

An elementwise operation, $x \rightarrow \varphi(x)$, where $\varphi(x) = (x + |x|)/2$. The layer transforms an input vector $\mathbf{x}$ into an output vector $\mathbf{y}$, specifically, $\mathbf{y} = \{\varphi(x_1), \varphi(x_2), \dots, \varphi(x_n)\}$. There are no parameters to be optimised.

AveragePool

An average pooling layer along one axis, with a pool size of 2 and a stride of 2. For an input vector $\mathbf{x} = \{x_1, x_2, \dots, x_n\}$ the output vector $\mathbf{y}$ is given by $0.5\{x_1+x_2, x_3+x_4, \dots\}$. There are no parameters to be optimised.

As an example, consider the vector $\mathbf{x}$ = {0.5, 0.5, 0.9, 2.0, 0.5, 2.0, 0.9, 0.5, 0.5}, then the average pooling with pool size of 2 and stride 2 will give:

y = { (0.5+0.5)/2,

(0.9+2.0)/2,
(0.5+2.0)/2,
(0.9+0.5)/2} = {0.5, 1.45, 1.25, 0.7}

Note that the last number of **x** is ignored here because the pooling is not valid.

GlobalAverage

A global averaging along one axis. For an input vector **x** = {x_1, x_2, …, x_n} the output scalar is average(x_1, x_2, x_3, …}. There are no parameters to be optimised.

MaxPool

A maximum pooling layer with a pool size of 3 and a stride of 2. For an input vector **x** = {x_1, x_2, …, x_n} the output vector **y** is given by {max(x_1, x_2, x_3), max(x_3, x_4, x_5), …}. There are no parameters to be optimised.

As an example, consider the vector **x** = {0.5, 0.5, 0.9, 2.0, 0.5, 2.0, 0.9, 0.5, 0.5}, then the maximum pooling with pool size of 3 and stride 2 will give:

y = { max(0.5, 0.5, 0.9),
max(0.9, 2.0, 0.5),
max(0.5, 2.0, 0.9),
max(0.9, 0.5, 0.5)} = {0.9, 2.0, 2.0, 0.9}

As for the averaging pooling, only valid outputs are calculated. Thus, when the size of the input vector, length(**x**), is odd, the output for this layer has a size of (length(**x**)-1)/2, while when length(**x**) is even, the output for this layer has a size of (length(**x**)-2)/2. Thus, in the present case length(**x**) = 9 and the size of the output is (length(x)-1)/2 = 4.

Norm

A batch-normalisation layer, which maintains the mean output close to 0 and the output standard deviation close to 1. For each channel of the input the normalisation is performed along the mini-batch dimension, **x**. Specifically for each channel the output **y** is **y** = γ (**x** – mean(**x**))/σ(**x**) + b, where σ(**x**) is the standard deviation of **x**. This layer has two parameters to be optimised per channel of the input, γ and b.

⊕

An elementwise addition layer. For two input vectors, **x** and **y**, of identical size, the output vector, **z**, is calculated as: **z** = {z_1, z_2, …, z_n} = {x_1+y_1, x_2+y_2, …, x_n+y_n}.

⊗

An elementwise multiplication layer. For two input vectors, **x** and **y**, of identical size, the output vector, **z**, is calculated as: **z** = {z_1, z_2, …, z_n} = {x_1*y_1, x_2*y_2, …, x_n*y_n}.

Python Code for Building DDN$_{TR}$

```
NP=65
FilterFactor=32
kernel=(6,1)

def build_model():
    #
    import tensorflow as tf
    #
    def dfh_gelu(x):
        return tf.add(tf.tanh(x), tf.math.scalar_mul( 0.02,x))
    def dfh_sigm(x):
        return tf.add(tf.math.sigmoid(x), tf.math.scalar_mul( 0.02,x))

    tf.keras.utils.get_custom_objects().update({'dfh_gelu': tf.keras.layers.Activation(dfh_gelu), 'dfh_sigm':
tf.keras.layers.Activation(dfh_sigm)})

    time_0   =  tf.keras.layers.Input( shape=(NP*2,), name='Input_time' )
    ipap_0   =  tf.keras.layers.Input( shape=(NP*2,), name='Input_ipap' )

    #
    xi = tf.expand_dims( ipap_0, axis=-1 ) # ( Batch, [real,imag] )
    hi = tf.expand_dims( time_0, axis=-1 )

    def lstm_module(filters):
        def inside(x):
            # x[0]: main   track
            # x[1]: memory track

            hidden_0_1 = tf.keras.layers.Dense( filters, activation=dfh_gelu, use_bias=True)( x[1] )

            hidden_1_1 = tf.keras.layers.Dense( filters, activation=dfh_sigm)(x[0])
            hidden_1_2 = tf.keras.layers.Dense( filters, activation=dfh_sigm)(x[0])
            hidden_1_3 = tf.keras.layers.Dense( filters, activation=dfh_gelu)(x[0])
            hidden_1_4 = tf.keras.layers.Dense( filters, activation=dfh_sigm)(x[0])

            hidden_2_1 = tf.keras.layers.multiply([hidden_1_1,hidden_0_1])
            hidden_2_2 = tf.keras.layers.multiply([hidden_1_2,hidden_1_3])
            #
            hidden_3_1 = tf.keras.layers.add( [hidden_2_1, hidden_2_2])
            #
            hidden_4_1 = tf.keras.layers.Activation(activation=dfh_gelu)( hidden_3_1 )
            hidden_4_2 = tf.keras.layers.multiply([hidden_4_1,hidden_1_4])
            #
            return hidden_4_2, hidden_3_1
        return inside

    def conv_layer(x_shp=4, y_shp=4, kernel=11, name=''):
        def inside(x):

            x1 =  tf.expand_dims( x, axis=-2)

            x2t = tf.keras.layers.Conv2D( FilterFactor, kernel_size=kernel, dilation_rate=[1,1], activation=dfh_gelu,
padding='valid', name='conv1d_x2t'+name )(x1)
            x2s = tf.keras.layers.Conv2D( FilterFactor, kernel_size=kernel, dilation_rate=[1,1], activation=dfh_sigm,
padding='valid', name='conv1d_x2s'+name )(x1)
            x2  = tf.keras.layers.Conv2DTranspose( FilterFactor*2, kernel_size=kernel, dilation_rate=[1,1],
padding='valid')(tf.math.multiply( x2t,x2s ))

            x3i = tf.keras.layers.ZeroPadding2D( ( (0,1*(kernel[0]-1)),(0,0)))(x2)
            x3t = tf.keras.layers.Conv2D( FilterFactor, kernel_size=kernel, dilation_rate=[2,1], activation=dfh_gelu,
padding='valid', name='conv1d_x3t'+name )(x3i)
            x3s = tf.keras.layers.Conv2D( FilterFactor, kernel_size=kernel, dilation_rate=[2,1], activation=dfh_sigm,
padding='valid', name='conv1d_x3s'+name )(x3i)
            x3  = tf.keras.layers.Conv2DTranspose( FilterFactor*2, kernel_size=kernel, dilation_rate=[1,1],
padding='valid')(tf.math.multiply( x3t, x3s ))
            #
            x4i = tf.keras.layers.ZeroPadding2D( ( (0,2*(kernel[0]-1)),(0,0)))(x3)
            x4t = tf.keras.layers.Conv2D( FilterFactor, kernel_size=kernel, dilation_rate=[3,1], activation=dfh_gelu,
padding='valid', name='conv1d_x4t'+name )(x4i)
            x4s = tf.keras.layers.Conv2D( FilterFactor, kernel_size=kernel, dilation_rate=[3,1], activation=dfh_sigm,
padding='valid', name='conv1d_x4s'+name )(x4i)
            x4  = tf.keras.layers.Conv2DTranspose( FilterFactor*2, kernel_size=kernel, dilation_rate=[1,1],
padding='valid')(tf.math.multiply( x4t, x4s ))

            x5i = tf.keras.layers.ZeroPadding2D( ( (0,3*(kernel[0]-1)),(0,0)))(x4)
            x5t = tf.keras.layers.Conv2D( FilterFactor, kernel_size=kernel, dilation_rate=[4,1], activation=dfh_gelu,
padding='valid', name='conv1d_x5t'+name )(x5i)
            x5s = tf.keras.layers.Conv2D( FilterFactor, kernel_size=kernel, dilation_rate=[4,1], activation=dfh_sigm,
padding='valid', name='conv1d_x5s'+name )(x5i)
            x5  = tf.keras.layers.Conv2DTranspose( FilterFactor*2, kernel_size=kernel, dilation_rate=[1,1],
padding='valid')(tf.math.multiply(x5t,x5s))
```

```
          x6i = tf.keras.layers.ZeroPadding2D( ( (0,5*(kernel[0]-1)),(0,0)))(x5)
          x6t = tf.keras.layers.Conv2D( FilterFactor, kernel_size=kernel, dilation_rate=[6,1],activation=dfh_gelu, padding='valid',
name='conv1d_x6t'+name )(x6i)
          x6s = tf.keras.layers.Conv2D( FilterFactor, kernel_size=kernel, dilation_rate=[6,1],activation=dfh_sigm,
padding='valid', name='conv1d_x6s'+name )(x6i)
          x6  = tf.keras.layers.Conv2DTranspose( FilterFactor*2, kernel_size=kernel, dilation_rate=[1,1],
padding='valid')(tf.math.multiply(x6t,x6s))

          x7i = tf.keras.layers.ZeroPadding2D( ( (0,7*(kernel[0]-1)),(0,0)))(x6)
          x7t = tf.keras.layers.Conv2D( FilterFactor, kernel_size=kernel, dilation_rate=[8,1],activation=dfh_gelu, padding='valid',
name='conv1d_x7t'+name )(x7i)
          x7s = tf.keras.layers.Conv2D( FilterFactor, kernel_size=kernel, dilation_rate=[8,1],activation=dfh_sigm,
padding='valid', name='conv1d_x7s'+name )(x7i)
          x7  = tf.keras.layers.Conv2DTranspose( FilterFactor*2, kernel_size=kernel, dilation_rate=[1,1],
padding='valid')(tf.math.multiply(x7t,x7s))
          #
          x8i = tf.keras.layers.ZeroPadding2D( ( (0,9*(kernel[0]-1)),(0,0)))(x7)
          x8t = tf.keras.layers.Conv2D( FilterFactor, kernel_size=kernel, dilation_rate=[10,1],activation=dfh_gelu,
padding='valid', name='conv1d_x8t'+name )(x8i)
          x8s = tf.keras.layers.Conv2D( FilterFactor, kernel_size=kernel, dilation_rate=[10,1],activation=dfh_sigm,
padding='valid', name='conv1d_x8s'+name )(x8i)
          x8  = tf.keras.layers.Conv2DTranspose( FilterFactor*2, kernel_size=kernel, dilation_rate=[1,1],
padding='valid')(tf.math.multiply(x8t,x8s))

          x9i = tf.keras.layers.ZeroPadding2D( ( (0,11*(kernel[0]-1)),(0,0)))(x8)
          x9t = tf.keras.layers.Conv2D( FilterFactor, kernel_size=kernel, dilation_rate=[12,1],activation=dfh_gelu,
padding='valid', name='conv1d_x9t'+name )(x9i)
          x9s = tf.keras.layers.Conv2D( FilterFactor, kernel_size=kernel, dilation_rate=[12,1],activation=dfh_sigm,
padding='valid', name='conv1d_x9s'+name )(x9i)
          x9  = tf.keras.layers.Conv2DTranspose( FilterFactor*2, kernel_size=kernel, dilation_rate=[1,1],
padding='valid')(tf.math.multiply(x9t,x9s))

          x10i = tf.keras.layers.ZeroPadding2D( ( (0,13*(kernel[0]-1)),(0,0)))(x9)
          x10t = tf.keras.layers.Conv2D( FilterFactor, kernel_size=kernel, dilation_rate=[14,1],activation=dfh_gelu,
padding='valid', name='conv1d_x10t'+name )(x10i)
          x10s = tf.keras.layers.Conv2D( FilterFactor, kernel_size=kernel, dilation_rate=[14,1],activation=dfh_sigm,
padding='valid', name='conv1d_x10s'+name )(x10i)
          x10  = tf.keras.layers.Conv2DTranspose( FilterFactor*2, kernel_size=kernel, dilation_rate=[1,1],
padding='valid')(tf.math.multiply(x10t,x10s))

          x15 = tf.keras.layers.Add()([x1,x2,x3,x4,x5,x6,x7,x8,x9,x10])

          return tf.squeeze( x15, axis=2)

        return inside

    def lstm_layer(x_shp=4, y_shp=4, kernel=11, name=''):
        def inside(x):

            x0 = conv_layer(x_shp=x_shp, y_shp=y_shp, kernel=kernel, name='x_'+name)(x[0])
            h0 = conv_layer(x_shp=x_shp, y_shp=y_shp, kernel=kernel, name='h_'+name)(x[1])
            #
            x00 = tf.keras.layers.Permute( (2,1))(x0)
            h00 = tf.keras.layers.Permute( (2,1))(h0)
            #
            x11, h11 = lstm_module( x_shp )( [ x00, h00] )
            #
            x1 = tf.keras.layers.Permute( (2,1))(x11)
            h1 = tf.keras.layers.Permute( (2,1))(h11)
            #
            # final transformation
            x0p = tf.keras.layers.add( [ x0, \
                    tf.keras.layers.multiply( [ \
                        tf.keras.layers.Dense( FilterFactor*2, activation=dfh_gelu)(x[1]),   \
                             tf.keras.layers.Dense( FilterFactor*2, activation=dfh_gelu)(x1) \
                                              ]) \
                                      ] )
            #
            h0p = tf.keras.layers.add( [ h0, \
                    tf.keras.layers.multiply( [ \
                        tf.keras.layers.Dense( FilterFactor*2, activation=dfh_gelu)(x[0]),   \
                             tf.keras.layers.Dense( FilterFactor*2, activation=dfh_gelu)(h1) \
                                              ]) \
                                      ] )

            return x0p,h0p
        return inside

    x1,h1 = lstm_layer(x_shp=2*NP,y_shp=1, kernel=kernel, name='_l1')([ xi,hi] )
    x2,h2 = lstm_layer(x_shp=2*NP,y_shp=1, kernel=kernel, name='_l2')([ x1,h1] )
    x3,h3 = lstm_layer(x_shp=2*NP,y_shp=1, kernel=kernel, name='_l3')([ x2,h2] )
    x4,h4 = lstm_layer(x_shp=2*NP,y_shp=1, kernel=kernel, name='_l4')([ x3,h3] )
    x5,h5 = lstm_layer(x_shp=2*NP,y_shp=1, kernel=kernel, name='_l5')([ x4,h4] )
    #
    xhs = tf.keras.layers.concatenate([x1,x2,x3,x4,x5,h1,h2,h3,h4,h5], axis=2)
```

```
xhf = tf.keras.layers.Dense( 1, activation=dfh_gelu)( xhs )
#
final_rshp  = tf.squeeze( tf.keras.layers.Activation('linear', dtype='float32')( xhf ), axis=-1 )
final_rshp  = tf.math.scalar_mul( tf.constant( NP, dtype=tf.dtypes.float32 ), final_rshp )
#
final = tf.keras.models.Model(inputs=(time_0, ipap_0), outputs=final_rshp)
#
final.compile(optimizer=tf.keras.optimizers.Adam(),
              loss='mse',
              metrics=['mse','mae'])
return final
```

Python Code for Building DDN_{CS}

```
def build_model_denseConv(filters=16):
    #
    import tensorflow as tf
    #

    def dense_block(x, filty, num_layers):
        stack = []
        x = alt_conv(x, filty)
        stack.append(x)

        for k in range(num_layers-1):
            if k>0:
                x = alt_conv(keras.layers.Concatenate()(stack), filty)
            else:
                x = alt_conv(x, filty)

            stack.append(x)

        return keras.layers.Concatenate()(stack)

    def alt_conv(x, filty):
        x = keras.layers.BatchNormalization()(x)
        x = keras.layers.ReLU()(x)
        x = keras.layers.Conv1D(4*filty, kernel_size=1, strides=1, padding = 'same')(x)

        x = keras.layers.BatchNormalization()(x)
        x = keras.layers.ReLU()(x)
        x = keras.layers.Conv1D(filty, kernel_size=3, strides=1, padding = 'same')(x)
        return x

    def transition(x):
        x = keras.layers.BatchNormalization()(x)
        x = keras.layers.ReLU()(x)
        x = keras.layers.AveragePooling1D(pool_size=2, strides = 2)(x)

        return x

    input =  keras.layers.Input(shape=[128,1])
    x = input
    x = keras.layers.Conv1D(filters, kernel_size=7, strides=1, padding = 'same')(x)
    x = keras.layers.MaxPool1D(pool_size=3, strides=2)(x)

    x = dense_block(x, filters, 6)
    x = transition(x)

    x = dense_block(x, filters, 12)
    x = transition(x)

    x = dense_block(x, filters, 32)
    x = transition(x)

    x = dense_block(x, filters, 32)
    x = keras.layers.BatchNormalization()(x)
    x = keras.layers.ReLU()(x)
    x = keras.layers.GlobalAveragePooling1D()(x)
    x = keras.layers.Dense(256, activation="relu")(x)
    fin_dens = keras.layers.Dense(6, activation="sigmoid")(x)
    fin_dens_reshape = keras.layers.Reshape((3, 2), input_shape=(6,))(fin_dens)
    #
    model = keras.Model(inputs=[input], outputs=[fin_dens_reshape])
    #
    model.compile(loss=unique_pairs_loss,
                optimizer=keras.optimizers.Adam(lr=0.33e-4),
                metrics=[unique_pairs_loss, conf_loss]
    )
    #
    return model
```
